# Circulating Endocannabinoids and Cognitive Function in Older Adults

**DOI:** 10.14336/AD.2024.1427

**Published:** 2024-12-20

**Authors:** Shiraz Vered, Alexa S. Beiser, Liron Sulimani, Sharon Sznitman, Saptaparni Ghosh, Gil M Lewitus, David Meiri, Sudha Seshadri, Galit Weinstein

**Affiliations:** ^1^School of Public Health, University of Haifa, Haifa 3498838, Israel.; ^2^Department of Neurology, Boston University Chobanian & Avedisian School of Medicine, Boston, MA 02118, USA.; ^3^Department of Biostatistics, Boston University School of Public Health, Boston, MA 02118, USA.; ^4^The Framingham Study, Framingham, MA 01702, USA.; ^5^The Kleifeld Laboratory, Department of Biology, Technion-Israel Institute of Technology, Haifa, 3200003, Israel.; ^6^The Laboratory of Cancer Biology and Natural Drug Discovery, Department of Biology, Technion-Israel Institute of Technology, Haifa 3200003, Israel.; ^7^Glenn Biggs Institute for Alzheimer's and Neurodegenerative Diseases, University of Texas Health Sciences Center, San Antonio, TX 78229, USA.

**Keywords:** endocannabinoids, cognitive function, sex, apolipoprotein ɛ4, brain aging, neurodegeneration

## Abstract

The role of endogenous cannabinoids (endocannabinoids; eCBs) in cognitive-related processes has been demonstrated in preclinical studies. However, observational studies are lacking. We examined the associations of multiple circulating eCBs and eCB-like molecules with cognitive function in a sample of dementia-free older adults. In this exploratory, cross-sectional study, serum levels of 44 eCBs were analyzed in 237 older participants of the Framingham Heart Study Offspring cohort who attended examination cycle 9 (2011-2014). Linear regression models were used to examine the associations of eCB levels with cognitive function while adjusting for potential confounders and correcting for multiple testing. Effect modification by sex and apolipoprotein ɛ4 (ApoEɛ4) was additionally examined. Participants’ mean age was 73.3±6.2y and 40% were men. After correction for multiple comparisons, increased levels of *linoleic acid*, *linolenic acid*, *oleic acid*, *oleoyl alanine* and *palmitoyl alanine* were associated with poorer executive function (B±SE=-0.0002±0.0001, p=0.002; B±SE=-0.0005±0.0001, p<0.001; B±SE=-0.0002±0.0001, p=0.003; B±SE=-0.74±0.25, p=0.003 and B±SE=-1.75±0.62, p=0.005, respectively). In addition, elevated levels of *linolenoyl amide* and *linoleoyl amide* were associated with poorer verbal memory (B±SE=-1.45±0.44, p=0.001 and B±SE=-0.16±0.05, p<0.001, respectively) and attention (B±SE=-0.12±0.04, p<0.001 and B±SE=-0.013±0.004, p<0.001, respectively). A significant interaction with sex was observed such that most of the above associations were present only among women. Furthermore, associations between several eCBs and perceptual organization were observed only among participants with ApoEɛ4 genotype. We identified novel eCB compounds that may be related to cognitive function. Validation of these findings is warranted and should consider sex and ApoEɛ4 interactions.

## INTRODUCTION

The endocannabinoid (eCB) system is a conserved widespread neuromodulatory system that is involved in many processes of the body. It is made up of receptors, turnover enzymes and endogenously produced cannabinoids (endocannabinoids; eCBs), which are small lipophilic signaling molecules [1]. The best studied eCBs, *2-Arachidonoyl glycerol (2-AG)* and *Arachidonoyl ethanolamide (AEA)*, bind directly to cannabinoid receptors, which are part of the endocannabinoidome (eCBome) [2]. In addition to *2-AG* and *AEA*, a broad range of molecules modulate or interact with the classical eCBs and are therefore considered part of the eCBome, even though they are not eCBs themselves [3]. These include, for example, lipid precursors such as linoleic (LA), oleic (OA) and palmitic (PA) acids, and other lipid mediators such as palmitoylethanolamide (PEA). Accumulating evidence, mainly from preclinical studies, stress the role of the eCBome in regulating multiple functions, including, neuroinflammation and neuro-degeneration [4]. Hence, the eCB system is considered a promising therapeutic target in general [5], and particularly for Alzheimer’s disease (AD) [6].

A plethora of preclinical studies highlights the role of the eCB system in cognitive function [7]. Indeed, it is known that the cannabinoid receptors are abundant in brain regions implicated in cognitive function [8]. Further, activation of these receptors by exogenous cannabinoids may result in short and long-term effects on cognitive function through modulation of the eCB system [9, 10]. Hence, the use of some eCB compounds, such as palmitoyl ethanolamide (*PEA*) [11], has been suggested as a promising therapeutic strategy to improve cognitive function, and ameliorate AD symptoms [12].

In a previous study from our group [13], we found significant associations between blood levels of several eCB compounds and markers of early neurodegeneration/neuro-injury. We additionally showed that these associations were largely modified by sex [13], a finding well supported by preclinical studies showing sex differences in the expression and activity of eCBs in the brain [14, 15]. Additional research also underscores the possible interaction of eCB signaling with apolipoprotein ɛ4 (ApoEɛ4) with respect to brain activity [16, 17].

Despite the plethora of evidence from animal models and human post-mortem brain samples, observational studies assessing the link between eCBs levels and neurodegeneration are scarce. In addition, prior studies were limited by small sample sizes and focused only on the few most-studied eCBs [18, 19]. Hence, in this cross-sectional analysis among dementia-free older adults, we explored the associations of 44 eCBs and eCB-like molecules with cognitive performance and tested for effect modification by sex and ApoEɛ4.

## MATERIALS AND METHODS

### Study sample

The Framingham Heart Study (FHS) was initiated in 1948 and is a single-site, longitudinal community-based cohort study [20]. Since the study was established, three generations of participants have enrolled. Over 5,000 children and children’s spouses of participants from the original cohort, constitutes the Offspring cohort, enrolled in 1971 and completed up to ten quadrennial examinations to date. Further information regarding the design and selection criteria was previously provided [21]. The current study included individuals of the Offspring cohort who participated in examination cycle 9 (2011-2014). From 736 eligible attendees, we randomly selected 250 participants, all adhering to the following inclusion criteria: 1) age 65 years or older; 2) without dementia, stroke or other crucial neurological diseases (e.g., head trauma, multiple sclerosis) at the time of the examination visit; 3) completed brain magnetic resonance imaging (MRI) following the clinical examination and 4) had dementia follow-up. Thirteen participants were excluded due to a diagnosis of dementia or stroke after the clinical examinations but prior to the cognitive function assessment. Therefore, 237 individuals were included in the final sample for the current analysis. Data were obtained under a protocol approved by the institutional review board of the Boston University Medical Center, and written informed consent was obtained from all participants.

### Circulating eCB levels

eCBs levels were tested from blood serum samples that were drawn at examination nine with participants fasting. Serum stored in the FHS laboratory at -80°C was shipped in 250 tubes of 0.2 mL on dry ice to the Laboratory of Cancer Biology and Natural Drug Discovery, Faculty of Biology, Technion-Israel Institute of Technology, Haifa, Israel. Additionally, the shipment included 14 phantom specimens (duplicated tubes that were blinded to the laboratory where the eCBs were measures) for assessment of measurement reliability. Acceptable accuracies for the vast majority of eCBs ranged between 90% and 110% and had less than 15% repeatability and reproducibility values. The temperature was monitored throughout the shipment using a temperature data logger. eCBs and eCB-like compounds were simultaneously extracted, identified and quantified in serum, via liquid chromatography high-resolution mass spectrometry (LC/HRMS). Information regarding the laboratory measures for circulating eCBs are described elsewhere [13]. We tested 58 eCBs in the serum. Values below the lowest detectable limit were set to this limit. Of the 58 eCBs, 14 were excluded because precise values for >90% measurements could not be obtained. Thus, serum levels of 44 eCBs were included in our analyses. Thirty-four eCBs were analyzed as continuous variables with normal distributions. Outliers were defined as ±4 standard deviation from the mean and excluded (<2% of values) because they were suspected to be measurements errors. The remaining 10 eCBs had at least 50% of their values below detectable levels and were therefore recoded as a binary variable with 0=lowest detectable value and 1=above detectable value. Supplementary Table 1 shows the full list of eCB compounds assessed in the current study along with their classification and abbreviations.

### Cognitive assessment

Study participants were administered a cognitive test battery by trained examiners using standard administration protocols [22]. The battery included the following five commonly used cognitive function tests: (1) verbal memory (WMS-III Logical Memory-Delayed Recall, score 0-24), (2) visual memory (WMS-III Visual Reproductions-Delayed Recall, score 0-14) (3) abstract reasoning (WAIS-III Similarities subtest, score 0-26), (4) perceptual organization (Hooper Visual Organization Test, score 0-30) and (5) attention and executive function (Trail-making Test A and Trail-making Test B minus A, respectively). Hooper Visual Organization Test and Trails making tests were log-transformed to normalize their distributions and transformed so that a higher score indicates better performance consistent with the other measures.

### Covariate ascertainment

All study covariates were measured at examination cycle 9. Demographic factors (age, sex, and education) were assessed via questionnaires. Body mass index (BMI) was calculated based on measurements of height and weight and obesity was defined as BMI>=30 kg/m^2^. ApoEɛ4 genotype was defined as the presence of at least one ApoE ɛ4 allele.

### Statistical analysis

Continuous variables were reported as mean and standard deviation or median (q1, q3) in case of a skewed distribution. Categorical variables were presented as frequency and percentage. Multivariable linear regression models were used to examine the association between each eCB compound and cognitive test scores. All models were adjusted for age, age squared (to account for nonlinear associations between age and cognition), sex, education level, ApoEɛ4 genotype, obesity and the time between the clinic examination (i.e., blood draw) and the cognitive examination. For each model, a p-value of 0.05 was used to indicate statistically significant results. In addition, the False Discovery Rate (FDR) method was used to correct for multiple comparisons in the total sample. Next, we examined whether sex and ApoEɛ4 genotype modified the associations between eCB levels and the study outcomes by including interaction terms of each eCB with these variables in the models. In cases where the interaction terms were statistically significant (defined as a p-value < 0.1 due to low power of these tests and the exploratory nature of the study), we ran stratified analyses by sex and ApoEɛ4 genotype. All statistical analyses were conducted using SAS version 9.4.

## RESULTS

The sample characteristics are presented in Table 1. Of the 237 participants, 95 (40%) were men and the mean age was 73.3±6.2 years. The mean time duration between the blood draw and the cognitive assessment was 1.6±1.0 years. Supplementary Table 2 shows the comparison of the main characteristics between the study sample (n=237) and Offspring cohort participants who attended exam nine and were eligible for the study but were not included in the study (n=709).

**Table 1:** Sample characteristics.

Variables	Overall N=237
Age, y	73.3 ±6.2
Sex (male)	95 (40.1)
Education (college)	165 (69.6)
Apolipoprotein ɛ4 genotype	46 (19.8)
Obesity	64 (27.0)
Time between blood draw and cognitive assessment, y	1.6 ±1.0
**Cognitive function**	
Verbal memory	11.3 ±3.9
Visual memory	6.5 ±3.0
Abstract reasoning	16.4 ±3.8
Perceptual organization	25.0 [23.0-27.0]
Attention	0.6 [0.5-0.7]
Executive function	0.9 [0.6-1.5]

Continuous traits values are reported as mean ±SD or median [Q1-Q3] and dichotomous traits values are reported as number (percent). Cognitive function: Verbal memory = Logical Memory-Delayed Recall test; Visual memory = Visual Reproductions-Delayed Recall test; Abstract reasoning = Similarities test; Perceptual organization = Hooper Visual Organization Test; Attention = Trail-making Test A; Executive function = Trail-making Test B minus A.

**Table 2:** Associations between endocannabinoids levels and cognitive function.

Endocannabinoid	Verbal memory			Visual memory			Abstract reasoning			Perceptual organization			Attention			Executive function		
	B (SE)	P _Raw_	P _FDR_	B (SE)	P _Raw_	P _FDR_	B (SE)	P _Raw_	P _FDR_	B (SE)	P _Raw_	P _FDR_	B (SE)	P _Raw_	P _FDR_	B (SE)	P _Raw_	P _FDR_
**Fatty Acids**																		
Linoleic acid (LA)	-0.002 (0.0009)	0.070	0.430	-0.0009 (0.0007)	0.173	0.851	-0.0005 (0.0008)	0.529	0.979	-0.0001 (0.0001)	0.582	0.988	0.0000 (0.0001)	0.964	0.964	-0.0002 (0.0001)	0.002	0.036
Linolenic acid (LnA)	-0.004 (0.0020)	0.029	0.321	-0.0020 (0.0010)	0.174	0.851	0.0002 (0.0020)	0.912	0.989	-0.0002 (0.0002)	0.292	0.988	0.0000 (0.0002)	0.862	0.943	-0.0005 (0.0001)	<0.001	0.022
Oleic acid (OA)	-0.001 (0.0006)	0.129	0.503	-0.0006 (0.0005)	0.182	0.851	-0.0002 (0.0006)	0.695	0.979	0.0000 (0.0001)	0.557	0.988	0.0000 (0.0001)	0.708	0.943	-0.0002 (0.0001)	0.003	0.036
**Fatty acid amides**																		
**Linolenoyl amide (Ln-Am)**	-1.45 (0.44)	0.001	0.026	-0.22 (0.33)	0.496	0.851	-0.24 (0.39)	0.540	0.979	0.005 (0.060)	0.932	0.988	-0.12 (0.040)	>0.001	0.018	-0.07 (0.040)	0.069	0.356
Linoleoyl amide (L-Am)	-0.16 (0.05)	<0.001	0.018	-0.02 (0.03)	0.474	0.851	0.02 (0.04)	0.662	0.979	0.004 (0.006)	0.519	0.988	-0.01 (0.004)	<0.001	0.018	-0.001 (0.004)	0.725	0.961
**N-Acyl Amino Acids**																		
**Oleoyl alanine (O-Ala)**	-7.27 (3.15)	0.022	0.321	-3.00 (2.36)	0.206	0.851	-3.84 (2.74)	0.163	0.899	-0.51 (0.38)	0.176	0.988	0.069 (0.26)	0.791	0.943	-0.74 (0.25)	0.003	0.036
**Palmitoyl alanine (P-Ala)**	-13.96 (7.77)	0.074	0.430	-9.35 (5.78)	0.107	0.851	-13.67 (6.79)	0.045	0.899	-1.20 (0.92)	0.198	0.988	0.32 (0.64)	0.614	0.943	-1.75 (0.62)	0.005	0.046

SE, Standard error; Bold values indicate statistical significance (p<0.05) after FDR correction for multiple comparisons Cognitive function: Verbal memory = Logical Memory-Delayed Recall test; Visual memory = Visual Reproductions-Delayed Recall test; Abstract reasoning = Similarities test; Perceptual organization = Hooper Visual Organization Test; Attention = Trail-making Test A; Executive function = Trail-making Test B minus A. Models adjusted for age, age squared, sex, education, apolipoprotein ɛ4 genotype, obesity and time between blood draw and cognitive assessment.

## Associations between eCBs levels and cognitive function

The associations between levels of each of the 44 eCB compounds and cognitive outcomes in the total sample are reported in Supplementary Table 3. Effect sizes with raw p-values and FDR-corrected p-values for the associations between eCBs and cognitive function in the total sample are presented in Table 2 for eCB compounds that were significantly associated (p_FDR_ <0.05) with at least one cognitive domain. Increased levels of the fatty acids *LA*, *linolenic acid (LnA)* and *OA*, and the N-acyl amino acids oleoyl *alanine (O-Ala)* and *palmitoyl alanine (P-Ala)* were associated with poorer executive function (B±SE=-0.0002±0.0001, p _FDR_=0.036; B±SE=-0.0005±0.0001, p_FDR_ =0.022 and B±SE=-0.0002±0.0001, p_FDR_ =0.036, B±SE=-0.74±0.25, p_FDR_ =0.036 and B±SE=-1.75±0.62, p_FDR_ =0.046, respectively). In addition, increased levels of the fatty acid amides *linolenoyl amide (Ln-Am)* and *linoleoyl amide (L-Am)* were associated with poorer performance on verbal memory (B±SE=-1.45±0.44, p_FDR_ =0.026 and B±SE=-0.16±0.05, p_FDR_ =0.018, respectively) and attention (B±SE=-0.12±0.04, p_FDR_ =0.018 and B±SE=-0.01±0.004, p_FDR_ 0.018, respectively).

## Associations by sex

Sample characteristics by sex are presented in Supplementary Table 4. The mean age of women and men was 73.6 ±6.6 and 72.8 ±5.5 years, respectively, and the mean time duration between the blood draw and cognitive assessment was 1.6 ±1.0 and 1.7 ±1.0 years, respectively. The interactions between all eCBs and sex in relation to cognitive assessment scores are reported in Supplementary Table 5.

**Figure 1.**
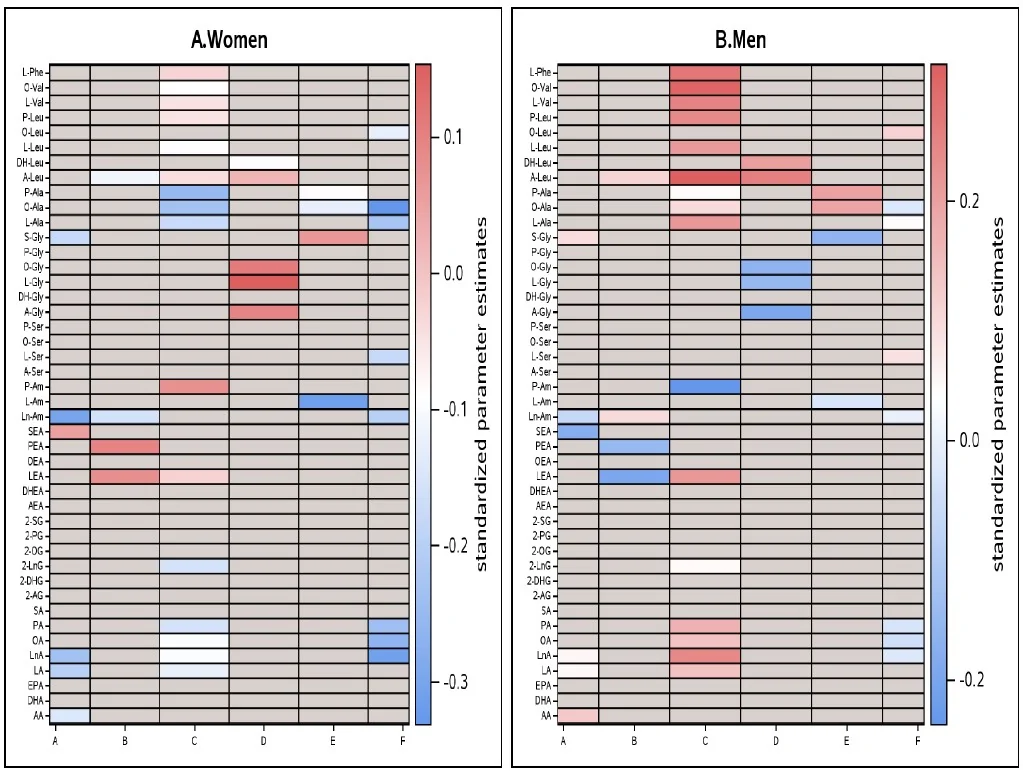
**Heatmaps indicating differences in the associations between endocannabinoid levels and cognitive function by sex**. Colored cells represent significant interactions between endocannabinoids and sex (P<0.1). Abbreviations of the cognitive function: A = verbal memory (Logical Memory-Delayed Recall test); B = visual memory (Visual Reproductions-Delayed Recall test); C = abstract reasoning (Similarities test); D = perceptual organization (Hooper Visual Organization Test); E = attention (Trail-making Test A); F = executive function (Trail-making Test B minus A). For abbreviations see Supplementary Table 1.

Figure 1 and Supplementary Table 6 depict the significant interactions between eCB levels and cognitive function in the various domains by sex. eCBs with at least one interaction with sex (P<0.1) are shown. The significant association between increased *LnA*, *OA* and *O-Ala* levels and poorer executive function in the total sample, was present in women (B±SE=-0.0007±0.0002, p<0.001, B±SE=-0.0002±0.0001, p=0.003 and B±SE=-1.36±0.37, p<0.001, respectively) but not men (B±SE=-0.0001±0.0002, p=0.821, B±SE=0.0001±0.0001, p=0.663 and B±SE=-0.079±0.34, p=0.817, respectively). Similarly, the association observed in the total sample, between increased *Ln-Am* levels and poorer verbal memory and between *L-Am* levels and poorer attention, were significant in women (B±SE=-2.01±0.56, p<0.001 and B±SE=-0.019±0.005, p<0.001, respectively) but not in men (B±SE=-0.44±0.74, p=0.554 and B±SE=-0.002±0.006, p=0.767, respectively).

Additional significant associations were observed when the models were stratified by sex. For example, high levels of *linoleoyl alanine* (*L-Ala*; N-acyl amino acids) were related to poor abstract reasoning in women, but to better abstract reasoning in men (B±SE=-45.49±22.48, p=0.045 and B±SE=43.67±20.13, p=0.033, respectively). Furthermore, high levels of several eCB compounds, also from the N-Acyl Amino Acids class, (e.g., *linoleoyl leucine (L-Leu)* and *palmitoyl leucine (P-Leu)*) were significantly associated with improved abstract reasoning in men only.

## Associations by ApoEɛ4

**Figure 2.**
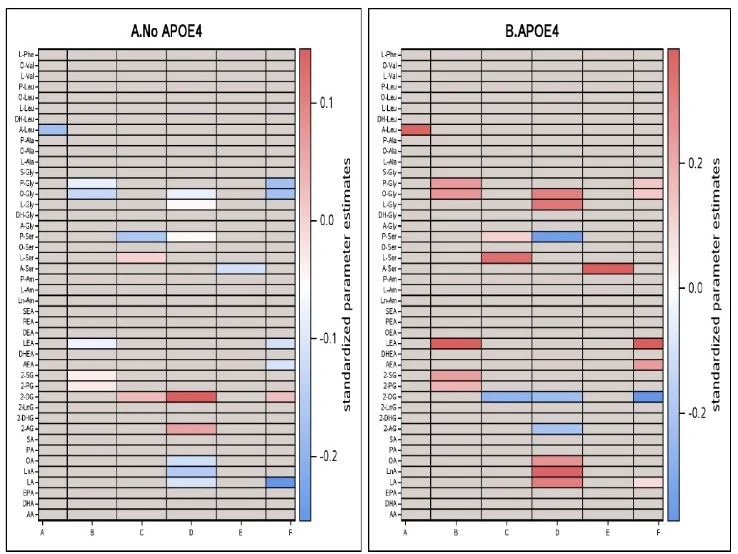
**Heatmaps indicating differences in the associations between endocannabinoid levels and cognitive function by apolipoprotein ɛ4 genotype**. Colored cells represent significant interactions between endocannabinoids and sex (P<0.1). Abbreviations: APOE4 = Apolipoprotein ɛ4 genotype. Abbreviations of the cognitive function: A = verbal memory (Logical Memory-Delayed Recall test); B = visual memory (Visual Reproductions-Delayed Recall test); C = abstract reasoning (Similarities test); D = perceptual organization (Hooper Visual Organization Test); E = attention (Trail-making Test A); F = executive function (Trail-making Test B minus A). For abbreviations see Supplementary Table 1

The main sample characteristics by ApoEɛ4 genotype status are presented in Supplementary Table 4. Mean age of those with no ApoEɛ4 genotype was 73.7 ±6.2 vs. 72.4 ±5.9 years for those with this genotype. The interactions between eCBs and ApoEɛ4 in relation to cognitive assessment scores are reported in Supplementary Table 5. Figure 2 and Supplementary Table 7 show the associations between eCB levels and cognitive function by ApoEɛ4 status. Only associations in which at least one significant interaction (P<0.1) between eCB and ApoEɛ4 was found are presented.

Stratification by ApoEɛ4 yielded new associations that were not apparent in the total sample. For example, no associations were observed with regard to perceptual organization performance in the total sample. However, in this stratified model we observed that among those with ApoEɛ4, but not among those without, the increased fatty acids *LA*, *LnA*, increased N-acyl amino acids *linoleoyl glycine (L-Gly)*, *oleoyl glycine (O-Gly)* and decreased N-acyl amino acids *palmitoyl serine (P-Ser)* levels were associated with better perceptual organization (B±SE=0.0007±0.0003, p=0.030; B±SE=0.002±0.0006, p=0.009; B±SE=1.89±0.81, p=0.025; B±SE=0.80±0.36, p=0.029 and B±SE=-1.47±0.62, p=0.024, respectively). In addition, increased N-acyl amino acid *arachidonoyl leucine (A-Leu)* was associated with better performance on verbal memory in participants with ApoEɛ4, but with poor performance on this domain in those without the ApoEɛ4 allele (B±SE=-1.42±0.63, p=0.027 and B±SE=3.02±1.25, p=0.021, respectively).

A summary of significant associations found in the study is presented in Table 3.

## DISCUSSION

Our exploration identified multiple circulating eCBs associated with cognitive function among community-dwelling, dementia-free older adults. Several eCBs are novel compounds with no previous information regarding their associations with cognitive health. Our study further underscores the importance of sex and the ApoEɛ4 as possible effect-modifiers, which should be taken into consideration in future studies of the eCBome and brain health.

The link between eCBs and cognitive function demonstrated in our study is supported by findings from multiple neuropathological, animal and cell culture studies. These studies highlighted several beneficial effects of eCBs including neuroprotection against excito-toxicity, inhibition of neuro-inflammation and neuro-degeneration, antioxidant activity and protection against Amyloid-Beta aggregation and toxicity [7, 23]. Yet, manipulations performed on the eCB system have shown contradictory effects on cognitive function, which are at least partly explained by excessive CB1 activation [24, 25].

Our findings suggest that increased circulating levels of the fatty acids *LA*, *LnA* and *OA* may be associated with poorer executive function. In general, fatty acids are essential players in central mechanisms underlying brain health including neurogenesis, neuronal inflammation, oxidative stress and neurotransmitter production [26]. Furthermore, fatty acid metabolism has been associated with AD pathology and cognitive function [27]. In line with our findings, a recent study showed that individuals with higher plasma levels of *LA*, the most abundant polyunsaturated n-6 fatty acid in the diet, had a faster cognitive decline [28]. This evidence raised the concern that excess levels of LA might adversely affect the brain due to its pro-inflammatory properties [29]. However, in contrast to our findings, other studies have shown that high circulating levels of LA may be related to lower risk of dementia and to improved cognitive function [30, 31]. Hence, it is postulated that LA may have neuroprotective effects, at least partly explained by its anti-oxidative properties, when not being used excessively [32]. Interestingly, we show that among carriers of the ApoEɛ4 genotype, high LA levels were related to better perceptual organization. Similarly, decreased stroke and mortality risk in relation to high plasma LA levels were previously observed only among FHS participants with, but not without, the ApoEɛ4 genotype [33]. These effect modifications by ApoEɛ4 can be explained by differential transport and metabolism of fatty acids based on ApoE ɛ4 status [34]. In addition to LA, high levels of *LnA*, a plant-derived n-3 fatty acid, have been associated in some prior literature with improved cardiometabolic health and anti-inflammatory effects [35, 36], while in our sample it was associated with poorer cognitive performance. However, most previous studies focused on LnA from diet rather than in blood, and its association with cognitive performance has been largely unexplored [36]. Lastly, OA, an n-9 fatty acid particularly abundant in olive oil is mostly known for its health benefits, including in the brain [37]. In addition, a recent study showed that high plasma levels of OA may be related to lower risk of mild cognitive impairment and AD [38-40]. The inconsistent findings comparing previous studies to the current research may stem from differences in study specious (i.e., cell cultures or animal models vs. humans), source of the fatty acids (e.g., diet, brain, CSF vs. blood), and differences in participants characteristics (e.g., age, disease/health status) [41]. Of note, the current associations of *LnA* and *OA* with executive function were found only in women. This finding is in accordance with previously noted interactions of fatty acids, particularly n-3 fatty acids, with sex with regard to cognition [40].

**Table 3:** Summary of significant associations.

Endocannabinoids	Total sample	By sex		By ApoEɛ4	
		Women	Men	No	Yes
Linoleic acid (LA)	Executive function ↓	Verbal memory ↓		Executive function ↓	Perceptual organization ↑
Linolenic acid (LnA)	Executive function ↓	Verbal memory ↓ Executive function ↓	Abstract reasoning ↑		Perceptual organization ↑
Oleic acid (OA)	Executive function ↓	Executive function ↓			
Palmitic acid (PA)		Abstract reasoning ↓ Executive function ↓			
2&1-Linolenoyl glycerol (2-LnG)		Abstract reasoning ↓			
2&1-Oleoyl glycerol (2-OG)					Executive function ↓
Linoleoyl ethanolamide (LEA)			Abstract reasoning ↑		Visual memory ↑ Executive function ↑
Linolenoyl amide (Ln-Am)	Verbal memory ↓ Attention ↓	Verbal memory ↓ Executive function ↓			
Linoleoyl amide (L-Am)	Verbal memory ↓ Attention ↓	Attention ↓			
Palmitoyl amide (P-Am)			Abstract reasoning ↑		
Oleoyl alanine (O-Ala)	Executive function ↓	Verbal memory ↓ Abstract reasoning ↓ Executive function ↓			
Palmitoyl alanine (P-Ala)	Executive function ↓	Abstract reasoning ↓			
Linoleoyl alanine (L-Ala)		Abstract reasoning ↓ Executive function ↓	Abstract reasoning ↑		
Arachidonoyl serine (A-Ser)					Perceptual organization ↓
Linoleoyl serine (L-Ser)		Executive function ↓			Abstract reasoning ↑
Palmitoyl serine (P-Ser)				Abstract reasoning ↓	Perceptual organization ↓
Arachidonoyl leucine (A-Leu)			Perceptual organization ↑	Verbal memory ↓	Verbal memory ↑
Linoleoyl leucine (L-Leu)			Abstract reasoning ↑		
Palmitoyl leucine (P-Leu)			Abstract reasoning ↑		
Linoleoyl valine (L-Val)			Abstract reasoning ↑		
Oleoyl valine (O-Val)			Abstract reasoning ↑		
Linoleoyl phenylalanine (L-Phe)			Abstract reasoning ↑		
Stearidonoyl glycine (S-Gly)		Verbal memory ↓			
Oleoyl glycine (O-Gly)				Executive function ↓	Perceptual organization ↑
Palmitoyl glycine (P-Gly)				Executive function ↓	
Linoleoyl glycine (L-Gly)					Perceptual organization ↑

Abbreviation: ApoEɛ4 = apolipoprotein ɛ4 genotype Cognitive function: Verbal memory = Logical Memory-Delayed Recall test; Visual memory = Visual Reproductions-Delayed Recall test; Abstract reasoning = Similarities test; Perceptual organization = Hooper Visual Organization Test; Attention = Trail-making Test A; Executive function = Trail-making Test B minus A; “↑” = positive associations between endocannabinoids and domains of cognitive function; “↓” = negative associations between endocannabinoids and domains of cognitive function. Models adjusted for age, age squared, sex, education, apolipoprotein ɛ4 genotype, obesity and time between blood draw and cognitive assessment. Total sample: significantly after FDR (p-value<0.05) By sex: sex stratification is shown only for models with significant eCBs*sex interaction (p-value<0.1) and significant association by sex (p-value<0.05). By ApoEɛ4: ApoEɛ4 stratification is shown only for models with significant eCBs*ApoEɛ4 interaction (p-value<0.1) and significant associations by ApoEɛ4 (p-value<0.05).

In general, fatty acid amides such *PEA* and *AEA* have consistently shown neuroprotective effects and a potential therapeutic effect for AD [42-45]. However, most research focused on the fatty acid ethanolamide class of fatty acid amides, while less is known on the association between fatty acid primary amides and brain health. Results from the current study does not support the evidence for neuroprotection of fatty acid ethanolamides, but rather suggest that high plasma levels of *Ln-Am* and *L-Am*, which are fatty acid primary amides, are associated with poorer verbal memory and attention. In agreement with these findings, a prior study that explored a panel of 883 plasma metabolites demonstrated a link of several fatty acid primary amides, including L-Am, with AD pathology and clinical diagnosis [46].

Another eCB class highlighted in our research is the N-acyl amino acids. N-acyl amino acids are eCBs that gained renewed scientific interest following the discovery of *AEA*, one of the most studied eCB compound, which binds directly to CB1 and CB2 receptors [47]. However, little is known to date on the molecular functions of the N-acyl amino acid family [47]. Specifically, our exploration showed that increased levels of N-acyl alanine (i.e., *O-Ala* and *P-Ala*) may be associated with poorer executive function. An *in vitro* exploration previously showed that compounds within the N-acyl alanine family may impair cell proliferation possibly through increased reactive oxygen species [48], a process that is altered in AD brains [49]. Our sex-stratified analyses identified additional N-acyl amino acids that are related to cognitive function, among men only. These findings are supported by a previous study from our group, demonstrating an association between eCBs from the N-acyl amino acid family with Glial fibrillary acidic protein (GFAP), a marker of neuronal injury, in men but not women [50].

Several biological explanations can be suggested for the sex interactions observed in our study. For example, the observation that the fatty acid LnA was associated with poorer executive function only among women may be explained by the decreased estrogen levels of the women in our sample, most of whom were at the postmenopausal phase. Indeed, estrogen may accelerate the transformation of LnA to Docosahexaenoic acid (*DHA*) and Eicosapentaenoic acid (*EPA*) [51, 52], which in turn, have shown associations with decreased AD risk [53, 54] and improved executive function [55, 56]. In addition, the fact that fatty acid amides were associated with poorer cognition only among women may be attributed to the strong influence of sex on fatty acid amide hydrolase (FAAH) [57, 58] which is responsible for the regulation of fatty acid amide levels [59]. Lastly, the observed effect modifications by ApoEɛ4 may be due to binding of ApoEɛ4 to sortilin receptor, which in turn disrupts sortilin’s ability to convert polyunsaturated fatty acid to eCB compounds [16].

Our study has several novelties and strengths. First, the study focused on many of the known eCB compounds, while others mostly addressed 3-5 specific compounds or single families [60]. Furthermore, the comprehensive cognitive assessment among community-dwelling dementia-free adults allows a valid generalizability to wide populations. Nevertheless, several limitations should be acknowledged. First, conclusions drawn from our study cannot infer a temporal relationship between eCBs levels and cognitive performance due to the study’s cross-sectional design. Second, the statistical power of our study is limited due to small sample sizes, particularly in the stratified analyses. Therefore, we consider our findings preliminary and encourage their validation in larger and diverse samples. Third, it is important to note the limited stability of eCB compounds. We acknowledged this limitation and therefore used the optimal laboratory practice (e.g., plasma frozen without delay, no repeated freeze/thaw cycles, blood draw in resting state after fasting). Fourth, little is known about the changes in eCBs levels over time in humans. Hence, changes in circulating eCB levels may have occurred in the time duration between the blood draw and the cognitive assessment. Fifth, it is unclear whether circulating eCBs levels reflect brain signaling. Yet, evidence shows that eCBs can cross the blood brain barrier [61]. Additionally, results showing that circulating eCB levels are linked with AD-related health conditions such as cardiovascular diseases, obesity and depression [24, 62] may support their role in cognition. Sixth, the external validity of our study is restricted, because it includes participants predominantly of high socioeconomic status and European ancestry and from one geographic region. Lastly, although main relevant covariates were considered, some residual confounding may remain. Particularly, lifestyle factors such as exercise and nutrition may confound the association between eCBs and cognitive since they have been associated with both cognitive function [63, 64] and eCBs levels [65, 66].

In conclusion, we demonstrate several significant associations between certain eCB molecules and cognitive function in dementia free middle- and old-age individuals who reside in the community. Overall, our findings support existing explorations in animal models and cell cultures, stressing the possible role of eCB signaling in cognitive performance. Yet, the variation in the direction of the eCB-cognition associations, together with the evidence of sex and ApoEɛ4 genotype highlight the complexity of the eCBome and the need for validations in external samples. A better understanding of the role of eCBs in cognitive health is important, since some eCB molecules may serve as blood-based biomarkers for cognitive impairment. Further, the levels of many eCB compounds including fatty acids, fatty acid amid and N-acyl amino acids may be modulated by interventions such as diet, hence may show therapeutic benefits for cognitive aging in older persons.

## Supplementary Materials


