# Supplementary material for: Circulating Endocannabinoids and Cognitive Function in Older Adults
Source: Aging Dis. 2024 Dec 20;17(1):518–29. doi: 10.14336/AD.2024.1427 (PMC12727136; doi:10.14336/AD.2024.1427)
Supplement: Supplementary file 1 [file AD-17-1-518-s.pdf]

Circulating Endocannabinoids and Cognitive Function in Older Adults

Shiraz Vered, Alexa S. Beiser, Liron Sulimani, Sharon Sznitman, Saptaparni Ghosh, Gil M Lewitus, David Meiri, Sudha Seshadri, Galit Weinstein

© 2024. Vered S. et al. Published online at http://www.aginganddisease.org/EN/10.14336/AD.2024.1427

Supplementary Table 1. list of the eCB compounds and their classification and abbreviations.

General family	Lipid class	Endocannabinoids
Fatty Acids	Fatty Acids (FAs)	Arachidonic acid (AA)<br>Docosahexaenoic acid (DHA)<br>Eicosapentaenoic acid (EPA)<br>Linoleic acid (LA)<br>Linolenic acid (LnA)<br>Oleic acid (OA)<br>Palmitic acid (PA)<br>Stearic acid (SA)
Monoglycerides	2-Monoacyl glycerols (2-MAGs)	2&1-Arachidonoyl glycerol (2-AG)<br>2&1-Docosahexaenoyl glycerol (2-DHG)<br>2&1-Linolenoyl glycerol (2-LnG)<br>2&1-Oleoyl glycerol (2-OG)<br>2&1-Palmitoyl glycerol (2-PG)<br>2&1-Steareoyl glycerol (2-SG)
Fatty acid amides	N-acyl ethanolamides (N-EAs) (Fatty acid ethanolamides)	Arachidonoyl ethanolamide (AEA)<br>Docosahexaenoyl ethanolamide (DHEA)<br>Linoleoyl ethanolamide (LEA)<br>Oleoyl ethanolamide (OEA)<br>Palmitoyl ethanolamide (PEA)<br>Stearoyl ethanolamide (SEA)
	N-acyl amides (N-Ams)<br>(Fatty acid primary amides)	N-Linolenoyl amide (Ln-Am)<br>N-Linoleoyl amide (L-Am)<br>N-Palmitoyl amide (P-Am)
N-Acyl Amino Acids	N-acyl serines (N-Sers)	N-Arachidonoyl serine (A-Ser)<br>N-Linoleoyl serine (L-Ser)<br>N-Oleoyl serine (O-Ser)<br>N-Palmitoyl serine (P-Ser)
	N-acyl glycines (N-Glys)	N-Arachidonoyl glycine (A-Gly)<br>N-Docosahexaenoyl glycine (DH-Gly)<br>N-Linoleoyl glycine (L-Gly)<br>N-Oleoyl glycine (O-Gly)<br>N-Palmitoyl glycine (P-Gly)<br>N-Stearidonoyl glycine (S-Gly)
	N-acyl alanines (N-Alas)	N-Linoleoyl alanine (L-Ala)<br>N-Oleoyl alanine (O-Ala)<br>N-Palmitoyl alanine (P-Ala)
	N-acyl leucines (N-Leus)	N-Arachidonoyl leucine (A-Leu)<br>N-Docosahexaenoyl leucine (DH-Leu)<br>N-Linoleoyl leucine (L-Leu)<br>N-Oleoyl leucine (O-Leu)<br>N-Palmitoyl leucine (P-Leu)
	N-acyl valines (N-Vals)	N-Linoleoyl valine (L-Val)<br>N-Oleoyl valine (O-Val)
	N-acyl phenylalanines (N-Phes)	N-Linoleoyl phenylalanine (L-Phe)

© 2024. Vered S. et al. Published online at http://www.aginganddisease.org/EN/10.14336/AD.2024.1427

Supplementary Table 2. Main characteristics of the study sample and participants not included in the analyses

Variables	Included random sample N=237	Not included* N=509
Age, y	73.3 ±6.2	72.8±5.8
Sex (men)	95 (40.1)	241 (47.4)
Education (college)	165 (69.6)	149 (29.3)
Apolipoprotein ε4 genotype	46 (19.8)	118 (23.9)
Obesity	64 (27.0)	157 (31.3)

Continuous values are reported as mean ±SD and dichotomous values are reported as N (%).
*Offspring cohort participants who attended exam nine but were not included in the study

© 2024. Vered S. et al. Published online at http://www.aginganddisease.org/EN/10.14336/AD.2024.1427

SUPPLEMENTARY DATA

Supplementary Table 3. Associations between levels of each of the 44 eCBs compounds and cognitive function in the total sample.

Endocannabinoids	Abbreviation	Outcomes	Crude model					Adjusted model				
			N	Parameter Estimate	Standard Error	raw P-value	FDR p-value	N	Parameter Estimate	Standard Error	raw P-value	FDR p-value
arachidonic acid	AA	Verbal memory	231	-0.0020	0.0034	0.5603	0.7953	226	-0.0020	0.0034	0.5621	0.8600
docosahexaenoic acid	DHA	Verbal memory	231	-0.0012	0.0035	0.7309	0.8675	226	-0.0031	0.0035	0.3749	0.7172
eicosapentaenoic acid	EPA	Verbal memory	229	0.0002	0.0073	0.9751	0.9751	224	-0.0019	0.0074	0.8021	0.8823
linoleic acid	LA	Verbal memory	231	-0.0015	0.0009	0.0863	0.3797	226	-0.0017	0.0009	0.0697	0.4296
linolenic acid	LnA	Verbal memory	231	-0.0029	0.0018	0.1030	0.3945	226	-0.0040	0.0018	0.0292	0.3212
oleic acid	OA	Verbal memory	231	-0.0008	0.0006	0.1807	0.5679	226	-0.0010	0.0006	0.1293	0.5031
palmitic acid	PA	Verbal memory	231	-0.0012	0.0012	0.3031	0.6351	226	-0.0007	0.0012	0.5536	0.8600
stearic acid	SA	Verbal memory	231	0.0003	0.0012	0.8044	0.9075	226	0.0015	0.0012	0.2054	0.5982
arachidonoyl ethanolamide	AEA	Verbal memory	230	-4.9454	4.5781	0.2812	0.6351	225	-3.1956	4.6372	0.4915	0.8600
docosahexaenoyl ethanolamide	DHEA	Verbal memory	231	-2.0984	2.8266	0.4586	0.7157	226	-3.1278	2.9219	0.2856	0.5984
linoleoyl ethanolamide	LEA	Verbal memory	230	-2.0348	2.7236	0.4558	0.7157	225	-0.7093	2.8188	0.8016	0.8823
oleoyl ethanolamide	OEA	Verbal memory	230	-1.7417	1.6259	0.2852	0.6351	225	-2.0127	1.7075	0.2398	0.5982
palmitoyl ethanolamide	PEA	Verbal memory	231	-1.1906	1.1291	0.2928	0.6351	226	-0.6797	1.1850	0.5668	0.8600
stearoyl ethanolamide	SEA	Verbal memory	231	-1.5047	1.7431	0.3889	0.7130	226	-0.7427	1.7844	0.6777	0.8770
docosahexaenoyl glycerol 2&1	2-DHG	Verbal memory	228	1.1255	0.8849	0.2047	0.6005	223	0.4499	0.8972	0.6166	0.8770
linolenoyl glycerol 2&1	2-LnG	Verbal memory	229	-0.1724	1.0435	0.8689	0.9103	225	-0.0577	1.0462	0.9561	0.9561
oleoyl glycerol 2&1	2-OG	Verbal memory	226	-0.0479	0.2173	0.8258	0.9084	222	0.0578	0.2178	0.7911	0.8823
palmitoyl glycerol 2&1	2-PG	Verbal memory	231	0.0240	0.0296	0.4184	0.7157	226	0.0322	0.0293	0.2719	0.5982
steareoyl glycerol 2&1	2-SG	Verbal memory	229	0.0050	0.0083	0.5496	0.7953	224	0.0083	0.0082	0.3098	0.6196
linoleoyl alanine	L-Ala	Verbal memory	230	-36.7372	16.1841	0.0241	0.1914	225	-25.5973	17.1107	0.1361	0.5031
oleoyl alanine	O-Ala	Verbal memory	230	-7.5252	3.0352	0.0139	0.1562	225	-7.2694	3.1499	0.0220	0.3212
palmitoyl alanine	P-Ala	Verbal memory	230	-16.5105	7.3705	0.0261	0.1914	225	-13.9633	7.7701	0.0737	0.4296
linolenoyl amide	Ln-Am	Verbal memory	228	-1.5846	0.4468	0.0005	0.0110	223	-1.4449	0.4414	0.0012	0.0264
linoleoyl amide	L-Am	Verbal memory	229	-0.1687	0.0463	0.0003	0.0110	224	-0.1623	0.0453	0.0004	0.0176
palmitoyl amide	P-Am	Verbal memory	229	-0.0547	0.0293	0.0633	0.3134	224	-0.0435	0.0292	0.1372	0.5031
linoleoyl glycine	L-Gly	Verbal memory	230	-4.6634	2.8864	0.1076	0.3945	225	-3.8958	2.9473	0.1876	0.5982
oleoyl glycine	O-Gly	Verbal memory	231	-2.4834	1.3348	0.0641	0.3134	226	-2.4989	1.4117	0.0781	0.4296
palmitoyl glycine	P-Gly	Verbal memory	231	-1.3712	0.7096	0.0545	0.3134	226	-1.2212	0.7380	0.0994	0.4860
linoleoyl leucine	L-Leu	Verbal memory	229	1.4489	4.5257	0.7492	0.8675	224	2.0411	4.8439	0.6739	0.8770
oleoyl leucine	O-Leu	Verbal memory	229	-0.6585	1.5078	0.6627	0.8675	224	-0.5461	1.5910	0.7318	0.8823
palmitoyl leucine	P-Leu	Verbal memory	230	-3.9535	9.9384	0.6911	0.8675	225	1.7256	10.8074	0.8733	0.9149
linoleoyl serine	L-Ser	Verbal memory	231	-5.6037	7.7728	0.4717	0.7157	226	-3.7543	7.8043	0.6310	0.8770
oleoyl serine	O-Ser	Verbal memory	230	-1.5897	2.9721	0.5933	0.8158	225	-1.9275	3.0705	0.5308	0.8600
palmitoyl serine	P-Ser	Verbal memory	231	-0.8117	1.9614	0.6794	0.8675	226	0.6322	2.0434	0.7573	0.8823
arachidonoyl serine	A-Ser	Verbal memory	231	-0.5119	0.5765	0.3755	0.7130	226	-0.1195	0.5770	0.8361	0.8973
linoleoyl phenylalanine	L-Phe	Verbal memory	231	0.6446	0.5476	0.2403	0.6220	226	0.6222	0.5507	0.2599	0.5982

© 2024. Vered S. et al. Published online at http://www.aginganddisease.org/EN/10.14336/AD.2024.1427

SUPPLEMENTARY DATA

arachidonoyl leucine	A-Leu	Verbal memory	231	-0.8182	0.5429	0.1331	0.4505	226	-0.6901	0.5678	0.2256	0.5982
docosahexaenoyl leucine	DH-Leu	Verbal memory	231	-0.5438	0.6106	0.3741	0.7130	226	-0.7704	0.6062	0.2051	0.5982
arachidonoyl glycine	A-Gly	Verbal memory	231	-1.2706	0.5143	0.0142	0.1562	226	-0.9764	0.5280	0.0658	0.4296
docosahexaenoyl glycine	DH-Gly	Verbal memory	231	0.1103	0.5897	0.8518	0.9103	226	0.2673	0.5865	0.6491	0.8770
stearidonoyl glycine	S-Gly	Verbal memory	231	-0.6310	0.5358	0.2401	0.6220	226	-0.6017	0.5407	0.2670	0.5982
linoleoyl valine	L-Val	Verbal memory	231	-0.4039	0.5228	0.4405	0.7157	226	-0.1771	0.5408	0.7436	0.8823
oleoyl valine	O-Val	Verbal memory	231	-0.0480	0.5746	0.9335	0.9552	226	-0.0727	0.5774	0.9000	0.9209
arachidonoyl glycerol 2&1	2-AG	Verbal memory	231	0.1783	0.5252	0.7346	0.8675	226	0.3635	0.5288	0.4926	0.8600
arachidonic acid	AA	Visual memory	232	-0.0030	0.0026	0.2452	0.8283	227	-0.0021	0.0026	0.4038	0.8513
docosahexaenoic acid	DHA	Visual memory	232	-0.0025	0.0027	0.3551	0.8283	227	-0.0031	0.0026	0.2458	0.8513
eicosapentaenoic acid	EPA	Visual memory	230	-0.0045	0.0056	0.4259	0.8283	225	-0.0044	0.0055	0.4295	0.8513
linoleic acid	LA	Visual memory	232	-0.0011	0.0007	0.1144	0.8283	227	-0.0009	0.0007	0.1733	0.8513
linolenic acid	LnA	Visual memory	232	-0.0021	0.0013	0.1221	0.8283	227	-0.0019	0.0014	0.1742	0.8513
oleic acid	OA	Visual memory	232	-0.0007	0.0005	0.1174	0.8283	227	-0.0006	0.0005	0.1820	0.8513
palmitic acid	PA	Visual memory	232	-0.0011	0.0009	0.2283	0.8283	227	-0.0007	0.0009	0.4390	0.8513
stearic acid	SA	Visual memory	232	0.0002	0.0009	0.8149	0.9194	227	0.0009	0.0009	0.2829	0.8513
arachidonoyl ethanolamide	AEA	Visual memory	231	0.6845	3.5058	0.8454	0.9287	226	1.6842	3.4910	0.6300	0.8939
docosahexaenoyl ethanolamide	DHEA	Visual memory	232	-1.8662	2.1486	0.3860	0.8283	227	-2.4647	2.1865	0.2609	0.8513
linoleoyl ethanolamide	LEA	Visual memory	231	-0.7838	2.0309	0.6999	0.9139	226	-0.3765	2.0743	0.8561	0.9659
oleoyl ethanolamide	OEA	Visual memory	230	0.3292	1.2305	0.7893	0.9139	225	0.5452	1.2795	0.6704	0.8939
palmitoyl ethanolamide	PEA	Visual memory	231	-0.2757	0.8621	0.7495	0.9139	226	0.2057	0.8880	0.8170	0.9460
stearoyl ethanolamide	SEA	Visual memory	231	0.1884	1.3502	0.8891	0.9287	226	1.2479	1.3492	0.3560	0.8513
docosahexaenoyl glycerol 2&1	2-DHG	Visual memory	229	-0.4631	0.6783	0.4954	0.9062	224	-0.6142	0.6738	0.3630	0.8513
linolenoyl glycerol 2&1	2-LnG	Visual memory	230	-0.6712	0.8128	0.4098	0.8283	226	-0.4801	0.7915	0.5448	0.8561
oleoyl glycerol 2&1	2-OG	Visual memory	227	-0.0603	0.1676	0.7195	0.9139	223	-0.0021	0.1626	0.9898	0.9997
palmitoyl glycerol 2&1	2-PG	Visual memory	232	0.0069	0.0225	0.7589	0.9139	227	0.0068	0.0220	0.7566	0.8997
steareoyl glycerol 2&1	2-SG	Visual memory	230	0.0024	0.0063	0.7048	0.9139	225	0.0023	0.0061	0.7142	0.8997
linoleoyl alanine	L-Ala	Visual memory	231	-14.7625	12.4113	0.2355	0.8283	226	-15.1228	12.7137	0.2355	0.8513
oleoyl alanine	O-Ala	Visual memory	231	-2.5174	2.3347	0.2820	0.8283	226	-3.0023	2.3643	0.2055	0.8513
palmitoyl alanine	P-Ala	Visual memory	231	-10.0860	5.6363	0.0749	0.8283	226	-9.3474	5.7805	0.1073	0.8513
linolenoyl amide	Ln-Am	Visual memory	229	-0.3126	0.3390	0.3574	0.8283	224	-0.2249	0.3295	0.4957	0.8513
linoleoyl amide	L-Am	Visual memory	230	-0.0280	0.0344	0.4166	0.8283	225	-0.0238	0.0332	0.4738	0.8513
palmitoyl amide	P-Am	Visual memory	230	-0.0332	0.0217	0.1283	0.8283	225	-0.0167	0.0213	0.4341	0.8513
linoleoyl glycine	L-Gly	Visual memory	231	-2.2450	2.2085	0.3105	0.8283	226	-1.7782	2.2261	0.4253	0.8513
oleoyl glycine	O-Gly	Visual memory	232	-1.0413	1.0119	0.3045	0.8283	227	-0.5599	1.0612	0.5983	0.8939
palmitoyl glycine	P-Gly	Visual memory	232	-0.1840	0.5470	0.7369	0.9139	227	-0.0002	0.5626	0.9997	0.9997
linoleoyl leucine	L-Leu	Visual memory	230	1.8487	3.4681	0.5945	0.9139	225	-0.2616	3.6424	0.9428	0.9997
oleoyl leucine	O-Leu	Visual memory	230	0.4496	1.1584	0.6983	0.9139	225	-0.5155	1.2007	0.6681	0.8939
palmitoyl leucine	P-Leu	Visual memory	231	11.2349	7.5996	0.1407	0.8283	226	5.8583	8.1301	0.4720	0.8513
linoleoyl serine	L-Ser	Visual memory	232	1.6000	5.9725	0.7890	0.9139	227	-0.0405	5.8974	0.9945	0.9997
oleoyl serine	O-Ser	Visual memory	231	-0.2217	2.2873	0.9229	0.9287	226	-0.8392	2.3464	0.7210	0.8997
palmitoyl serine	P-Ser	Visual memory	232	-1.4090	1.5067	0.3507	0.8283	227	-1.5863	1.5514	0.3077	0.8513
arachidonoyl serine	A-Ser	Visual memory	232	0.2186	0.4433	0.6224	0.9139	227	0.3042	0.4320	0.4821	0.8513

© 2024. Vered S. et al. Published online at http://www.aginganddisease.org/EN/10.14336/AD.2024.1427

linoleoyl phenylalanine	L-Phe	Visual memory	232	0.2602	0.4193	0.5355	0.9062	227	0.2805	0.4141	0.4989	0.8513
arachidonoyl leucine	A-Leu	Visual memory	232	0.2577	0.4152	0.5353	0.9062	227	-0.0360	0.4257	0.9326	0.9997
docosahexaenoyl leucine	DH-Leu	Visual memory	232	-0.3692	0.4701	0.4330	0.8283	227	-0.3789	0.4588	0.4097	0.8513
arachidonoyl glycine	A-Gly	Visual memory	232	-0.8602	0.3933	0.0298	0.8283	227	-0.6938	0.3969	0.0819	0.8513
docosahexaenoyl glycine	DH-Gly	Visual memory	232	-0.1709	0.4506	0.7049	0.9139	227	-0.2822	0.4405	0.5224	0.8513
stearidonoyl glycine	S-Gly	Visual memory	232	0.0365	0.4074	0.9287	0.9287	227	-0.1292	0.4021	0.7483	0.8997
linoleoyl valine	L-Val	Visual memory	232	0.4003	0.3990	0.3168	0.8283	227	0.2689	0.4037	0.5061	0.8513
oleoyl valine	O-Val	Visual memory	232	0.5048	0.4358	0.2479	0.8283	227	0.2960	0.4320	0.4940	0.8513
arachidonoyl glycerol 2&1	2-AG	Visual memory	232	-0.0604	0.3998	0.8800	0.9287	227	0.1717	0.3947	0.6641	0.8939
arachidonic acid	AA	Abstract reasoning	235	-0.0008	0.0032	0.7928	0.9459	230	0.0000	0.0030	0.9887	0.9887
docosahexaenoic acid	DHA	Abstract reasoning	234	0.0023	0.0034	0.5037	0.8368	229	-0.0001	0.0032	0.9860	0.9887
eicosapentaenoic acid	EPA	Abstract reasoning	232	0.0043	0.0071	0.5438	0.8545	227	0.0010	0.0067	0.8830	0.9887
linoleic acid	LA	Abstract reasoning	235	-0.0008	0.0008	0.3761	0.8368	230	-0.0005	0.0008	0.5293	0.9791
linolenic acid	LnA	Abstract reasoning	235	-0.0001	0.0017	0.9584	0.9584	230	0.0002	0.0016	0.9124	0.9887
oleic acid	OA	Abstract reasoning	235	-0.0004	0.0006	0.5135	0.8368	230	-0.0002	0.0006	0.6948	0.9791
palmitic acid	PA	Abstract reasoning	235	-0.0013	0.0011	0.2302	0.8368	230	-0.0008	0.0011	0.4462	0.9791
stearic acid	SA	Abstract reasoning	235	-0.0015	0.0011	0.1732	0.8368	230	-0.0007	0.0010	0.5028	0.9791
arachidonoyl ethanolamide	AEA	Abstract reasoning	234	-1.0038	4.4083	0.8201	0.9496	229	3.7152	4.1743	0.3744	0.8988
docosahexaenoyl ethanolamide	DHEA	Abstract reasoning	235	2.7370	2.6935	0.3106	0.8368	230	0.9693	2.6234	0.7121	0.9791
linoleoyl ethanolamide	LEA	Abstract reasoning	234	0.5003	2.5629	0.8454	0.9538	229	2.3551	2.4814	0.3436	0.8988
oleoyl ethanolamide	OEA	Abstract reasoning	233	-0.2594	1.5575	0.8679	0.9547	228	-0.0588	1.5382	0.9696	0.9887
palmitoyl ethanolamide	PEA	Abstract reasoning	234	-0.7037	1.0721	0.5122	0.8368	229	0.2880	1.0523	0.7846	0.9887
stearoyl ethanolamide	SEA	Abstract reasoning	234	0.1628	1.6717	0.9225	0.9584	229	1.5170	1.6059	0.3459	0.8988
docosahexaenoyl glycerol 2&1	2-DHG	Abstract reasoning	232	0.0503	0.8497	0.9528	0.9584	227	-0.3942	0.8045	0.6247	0.9791
linolenoyl glycerol 2&1	2-LnG	Abstract reasoning	233	-1.8585	1.0094	0.0669	0.4513	229	-1.2819	0.9416	0.1748	0.8988
oleoyl glycerol 2&1	2-OG	Abstract reasoning	230	-0.2528	0.2107	0.2314	0.8368	226	-0.1108	0.1959	0.5724	0.9791
palmitoyl glycerol 2&1	2-PG	Abstract reasoning	235	0.0073	0.0280	0.7954	0.9459	230	0.0028	0.0261	0.9145	0.9887

© 2024. Vered S. et al. Published online at http://www.aginganddisease.org/EN/10.14336/AD.2024.1427

steareoyl glycerol 2&1	2-SG	Abstract reasoning	233	0.0057	0.0077	0.4559	0.8368	228	0.0066	0.0071	0.3525	0.8988
linoleoyl alanine	L-Ala	Abstract reasoning	234	-17.3043	15.6476	0.2699	0.8368	229	-0.3492	15.2867	0.9818	0.9887
oleoyl alanine	O-Ala	Abstract reasoning	234	-5.7386	2.8476	0.0450	0.4513	229	-3.8351	2.7391	0.1629	0.8988
palmitoyl alanine	P-Ala	Abstract reasoning	234	-20.7130	6.9283	0.0031	0.1276	229	-13.6675	6.7887	0.0453	0.8988
linolenoyl amide	Ln-Am	Abstract reasoning	232	-0.3609	0.4259	0.3977	0.8368	227	-0.2429	0.3957	0.5400	0.9791
linoleoyl amide	L-Am	Abstract reasoning	233	0.0149	0.0433	0.7307	0.9459	228	0.0174	0.0399	0.6621	0.9791
palmitoyl amide	P-Am	Abstract reasoning	233	-0.0285	0.0277	0.3044	0.8368	228	-0.0119	0.0259	0.6460	0.9791
linoleoyl glycine	L-Gly	Abstract reasoning	234	0.7316	2.7826	0.7928	0.9459	229	1.6569	2.6644	0.5347	0.9791
oleoyl glycine	O-Gly	Abstract reasoning	235	-0.7283	1.2704	0.5670	0.8603	230	-0.2462	1.2579	0.8450	0.9887
palmitoyl glycine	P-Gly	Abstract reasoning	235	-0.3429	0.6896	0.6195	0.8793	230	-0.0421	0.6733	0.9502	0.9887
linoleoyl leucine	L-Leu	Abstract reasoning	233	3.4023	4.3243	0.4322	0.8368	228	3.8589	4.3506	0.3761	0.8988
oleoyl leucine	O-Leu	Abstract reasoning	233	1.8456	1.4334	0.1992	0.8368	228	1.6105	1.4200	0.2580	0.8988
palmitoyl leucine	P-Leu	Abstract reasoning	234	7.4086	9.4694	0.4348	0.8368	229	9.0630	9.6376	0.3481	0.8988
linoleoyl serine	L-Ser	Abstract reasoning	235	5.5053	7.4693	0.4618	0.8368	230	6.0684	7.0171	0.3881	0.8988
oleoyl serine	O-Ser	Abstract reasoning	234	0.3297	2.7790	0.9057	0.9584	229	0.3421	2.6825	0.8986	0.9887
palmitoyl serine	P-Ser	Abstract reasoning	235	-3.8559	1.8627	0.0396	0.4513	230	-3.3793	1.8258	0.0655	0.8988
arachidonoyl serine	A-Ser	Abstract reasoning	235	-1.0689	0.5482	0.0524	0.4513	230	-0.5550	0.5159	0.2832	0.8988
linoleoyl phenylalanine	L-Phe	Abstract reasoning	235	0.5017	0.5214	0.3369	0.8368	230	0.6223	0.4921	0.2074	0.8988
arachidonoyl leucine	A-Leu	Abstract reasoning	235	0.4307	0.5184	0.4070	0.8368	230	0.4853	0.5050	0.3376	0.8988
docosahexaenoyl leucine	DH-Leu	Abstract reasoning	235	0.2299	0.5846	0.6945	0.9459	230	0.1836	0.5458	0.7369	0.9825
arachidonoyl glycine	A-Gly	Abstract reasoning	235	-0.1391	0.4981	0.7803	0.9459	230	0.2551	0.4757	0.5923	0.9791
docosahexaenoyl glycine	DH-Gly	Abstract reasoning	235	1.0079	0.5573	0.0718	0.4513	230	0.7674	0.5204	0.1418	0.8988
stearidonoyl glycine	S-Gly	Abstract reasoning	235	1.3998	0.5024	0.0058	0.1276	230	0.9801	0.4776	0.0413	0.8988

© 2024. Vered S. et al. Published online at http://www.aginganddisease.org/EN/10.14336/AD.2024.1427

SUPPLEMENTARY DATA

linoleoyl valine	L-Val	Abstract reasoning	235	0.2700	0.5002	0.5898	0.8650	230	0.4307	0.4830	0.3734	0.8988
oleoyl valine	O-Val	Abstract reasoning	235	0.6668	0.5457	0.2230	0.8368	230	0.5103	0.5140	0.3219	0.8988
arachidonoyl glycerol 2&1	2-AG	Abstract reasoning	235	-0.3759	0.5016	0.4543	0.8368	230	0.1761	0.4760	0.7118	0.9791
arachidonic acid	AA	Perceptual organization	220	0.0002	0.0004	0.6450	0.9675	216	0.0002	0.0004	0.5537	0.9875
docosahexaenoic acid	DHA	Perceptual organization	219	0.0001	0.0005	0.8443	0.9675	215	-0.0001	0.0004	0.8237	0.9875
eicosapentaenoic acid	EPA	Perceptual organization	217	0.0004	0.0010	0.6828	0.9675	213	0.0001	0.0009	0.8805	0.9875
linoleic acid	LA	Perceptual organization	220	-0.0001	0.0001	0.5047	0.9675	216	-0.0001	0.0001	0.5818	0.9875
linolenic acid	LnA	Perceptual organization	220	-0.0002	0.0002	0.3997	0.9675	216	-0.0002	0.0002	0.2921	0.9875
oleic acid	OA	Perceptual organization	220	-0.0001	0.0001	0.4178	0.9675	216	0.0000	0.0001	0.5571	0.9875
palmitic acid	PA	Perceptual organization	220	-0.0002	0.0001	0.1802	0.9675	216	-0.0001	0.0001	0.5256	0.9875
stearic acid	SA	Perceptual organization	220	-0.0002	0.0001	0.1388	0.9675	216	-0.0001	0.0001	0.5932	0.9875
arachidonoyl ethanolamide	AEA	Perceptual organization	219	0.5687	0.5556	0.3071	0.9675	215	0.8704	0.5531	0.1171	0.9875
docosahexaenoyl ethanolamide	DHEA	Perceptual organization	220	0.0918	0.3560	0.7967	0.9675	216	0.1024	0.3624	0.7778	0.9875
linoleoyl ethanolamide	LEA	Perceptual organization	219	-0.1957	0.3359	0.5608	0.9675	215	0.0054	0.3455	0.9875	0.9875
oleoyl ethanolamide	OEA	Perceptual organization	219	-0.0307	0.2019	0.8795	0.9675	215	0.0237	0.2081	0.9096	0.9875
palmitoyl ethanolamide	PEA	Perceptual organization	220	-0.1482	0.1397	0.2901	0.9675	216	-0.0260	0.1438	0.8569	0.9875
stearoyl ethanolamide	SEA	Perceptual organization	220	-0.0574	0.2281	0.8015	0.9675	216	0.1128	0.2290	0.6229	0.9875
docosahexaenoyl glycerol 2&1	2-DHG	Perceptual organization	218	0.0890	0.1081	0.4114	0.9675	214	0.0195	0.1078	0.8567	0.9875
linolenoyl glycerol 2&1	2-LnG	Perceptual organization	218	-0.1432	0.1309	0.2750	0.9675	215	-0.1527	0.1287	0.2368	0.9875
oleoyl glycerol 2&1	2-OG	Perceptual organization	215	0.0179	0.0274	0.5142	0.9675	212	0.0299	0.0269	0.2666	0.9875
palmitoyl glycerol 2&1	2-PG	Perceptual organization	220	-0.0035	0.0036	0.3285	0.9675	216	-0.0033	0.0035	0.3542	0.9875
steareoyl glycerol 2&1	2-SG	Perceptual organization	218	-0.0002	0.0010	0.8645	0.9675	214	0.0001	0.0010	0.9488	0.9875
linoleoyl alanine	L-Ala	Perceptual organization	219	-2.0209	2.0489	0.3251	0.9675	215	-0.7674	2.1203	0.7178	0.9875

© 2024. Vered S. et al. Published online at http://www.aginganddisease.org/EN/10.14336/AD.2024.1427

oleoyl alanine	O-Ala	Perceptual organization	219	-0.5607	0.3732	0.1345	0.9675	215	-0.5155	0.3798	0.1762	0.9875
palmitoyl alanine	P-Ala	Perceptual organization	219	-1.6922	0.9011	0.0617	0.9049	215	-1.1976	0.9269	0.1978	0.9875
linolenoyl amide	Ln-Am	Perceptual organization	217	0.0004	0.0620	0.9949	0.9949	213	0.0052	0.0609	0.9321	0.9875
linoleoyl amide	L-Am	Perceptual organization	218	0.0036	0.0057	0.5208	0.9675	214	0.0035	0.0055	0.5194	0.9875
palmitoyl amide	P-Am	Perceptual organization	218	-0.0039	0.0036	0.2775	0.9675	214	-0.0028	0.0035	0.4290	0.9875
linoleoyl glycine	L-Gly	Perceptual organization	219	0.0260	0.3636	0.9430	0.9949	215	0.1653	0.3636	0.6499	0.9875
oleoyl glycine	O-Gly	Perceptual organization	220	-0.0575	0.1664	0.7301	0.9675	216	0.0366	0.1716	0.8312	0.9875
palmitoyl glycine	P-Gly	Perceptual organization	220	0.0180	0.0888	0.8397	0.9675	216	0.0798	0.0902	0.3773	0.9875
linoleoyl leucine	L-Leu	Perceptual organization	218	-0.2580	0.5617	0.6464	0.9675	214	-0.2092	0.5978	0.7268	0.9875
oleoyl leucine	O-Leu	Perceptual organization	218	-0.1148	0.1854	0.5366	0.9675	214	-0.0884	0.1937	0.6487	0.9875
palmitoyl leucine	P-Leu	Perceptual organization	219	-0.9035	1.2194	0.4595	0.9675	215	-0.4776	1.3081	0.7154	0.9875
linoleoyl serine	L-Ser	Perceptual organization	220	0.2836	0.9735	0.7711	0.9675	216	0.4939	0.9649	0.6093	0.9875
oleoyl serine	O-Ser	Perceptual organization	219	-0.1583	0.3747	0.6732	0.9675	215	-0.1079	0.3779	0.7755	0.9875
palmitoyl serine	P-Ser	Perceptual organization	220	-0.4817	0.2454	0.0509	0.9049	216	-0.3445	0.2544	0.1772	0.9875
arachidonoyl serine	A-Ser	Perceptual organization	220	-0.0021	0.0714	0.9768	0.9949	216	0.0398	0.0703	0.5722	0.9875
linoleoyl phenylalanine	L-Phe	Perceptual organization	220	0.0470	0.0686	0.4945	0.9675	216	0.0416	0.0688	0.5461	0.9875
arachidonoyl leucine	A-Leu	Perceptual organization	220	0.0885	0.0682	0.1960	0.9675	216	0.0999	0.0701	0.1554	0.9875
docosahexaenoyl leucine	DH-Leu	Perceptual organization	220	0.0373	0.0772	0.6291	0.9675	216	0.0258	0.0762	0.7357	0.9875
arachidonoyl glycine	A-Gly	Perceptual organization	220	-0.0740	0.0650	0.2564	0.9675	216	-0.0243	0.0651	0.7098	0.9875
docosahexaenoyl glycine	DH-Gly	Perceptual organization	220	-0.0327	0.0733	0.6560	0.9675	216	-0.0197	0.0719	0.7844	0.9875
stearidonoyl glycine	S-Gly	Perceptual organization	220	0.1300	0.0660	0.0502	0.9049	216	0.1330	0.0658	0.0445	0.9875
linoleoyl valine	L-Val	Perceptual organization	220	-0.0337	0.0651	0.6051	0.9675	216	-0.0128	0.0665	0.8478	0.9875
oleoyl valine	O-Val	Perceptual organization	220	0.0008	0.0718	0.9911	0.9949	216	0.0023	0.0714	0.9745	0.9875

© 2024. Vered S. et al. Published online at http://www.aginganddisease.org/EN/10.14336/AD.2024.1427

SUPPLEMENTARY DATA

arachidonoyl glycerol 2&1	2-AG	Perceptual organization	220	-0.0199	0.0660	0.7635	0.9675	216	0.0026	0.0661	0.9691	0.9875
arachidonic acid	AA	Attention	230	0.0004	0.0003	0.1939	0.7110	226	0.0005	0.0003	0.0701	0.7319
docosahexaenoic acid	DHA	Attention	229	0.0000	0.0003	0.9521	0.9742	225	0.0000	0.0003	0.8873	0.9433
eicosapentaenoic acid	EPA	Attention	227	0.0002	0.0006	0.7973	0.9028	223	0.0004	0.0006	0.5644	0.9433
linoleic acid	LA	Attention	230	0.0000	0.0001	0.6135	0.9028	226	0.0000	0.0001	0.9637	0.9637
linolenic acid	LnA	Attention	230	0.0000	0.0001	0.7532	0.9028	226	0.0000	0.0002	0.8618	0.9433
oleic acid	OA	Attention	230	0.0000	0.0001	0.4231	0.9028	226	0.0000	0.0001	0.7079	0.9433
palmitic acid	PA	Attention	230	-0.0001	0.0001	0.3363	0.9028	226	0.0000	0.0001	0.7580	0.9433
stearic acid	SA	Attention	230	0.0000	0.0001	0.8162	0.9028	226	0.0001	0.0001	0.2263	0.9433
arachidonoyl ethanolamide	AEA	Attention	229	-0.2032	0.3950	0.6075	0.9028	225	0.0661	0.3985	0.8684	0.9433
docosahexaenoyl ethanolamide	DHEA	Attention	230	-0.4738	0.2351	0.0450	0.4950	226	-0.4039	0.2444	0.0998	0.7319
linoleoyl ethanolamide	LEA	Attention	229	-0.3394	0.2254	0.1335	0.7092	225	-0.1629	0.2338	0.4866	0.9433
oleoyl ethanolamide	OEA	Attention	228	-0.1851	0.1368	0.1773	0.7092	224	-0.1424	0.1443	0.3247	0.9433
palmitoyl ethanolamide	PEA	Attention	229	-0.1304	0.0963	0.1770	0.7092	225	-0.0634	0.1004	0.5283	0.9433
stearoyl ethanolamide	SEA	Attention	229	-0.2368	0.1506	0.1173	0.7092	225	-0.1442	0.1534	0.3484	0.9433
docosahexaenoyl glycerol 2&1	2-DHG	Attention	227	0.1063	0.0737	0.1506	0.7092	223	0.0840	0.0747	0.2625	0.9433
linolenoyl glycerol 2&1	2-LnG	Attention	228	0.1470	0.0895	0.1019	0.7092	225	0.1570	0.0884	0.0772	0.7319
oleoyl glycerol 2&1	2-OG	Attention	225	0.0067	0.0186	0.7194	0.9028	222	0.0132	0.0183	0.4725	0.9433
palmitoyl glycerol 2&1	2-PG	Attention	230	0.0006	0.0025	0.8081	0.9028	226	0.0015	0.0024	0.5518	0.9433
steareoyl glycerol 2&1	2-SG	Attention	228	-0.0002	0.0007	0.8141	0.9028	224	0.0001	0.0007	0.8577	0.9433
linoleoyl alanine	L-Ala	Attention	230	-0.7372	1.3749	0.5924	0.9028	226	0.5987	1.4337	0.6767	0.9433
oleoyl alanine	O-Ala	Attention	230	-0.0578	0.2520	0.8188	0.9028	226	0.0686	0.2579	0.7905	0.9433
palmitoyl alanine	P-Ala	Attention	230	-0.0902	0.6185	0.8841	0.9467	226	0.3241	0.6407	0.6135	0.9433
linolenoyl amide	Ln-Am	Attention	227	-0.1399	0.0365	0.0002	0.0044	223	-0.1236	0.0363	0.0008	0.0176
linoleoyl amide	L-Am	Attention	228	-0.0140	0.0038	0.0002	0.0044	224	-0.0132	0.0037	0.0005	0.0176
palmitoyl amide	P-Am	Attention	228	-0.0054	0.0025	0.0298	0.4371	224	-0.0041	0.0025	0.0993	0.7319
linoleoyl glycine	L-Gly	Attention	229	-0.2944	0.2452	0.2311	0.7822	225	-0.1157	0.2507	0.6449	0.9433
oleoyl glycine	O-Gly	Attention	230	-0.1182	0.1120	0.2925	0.9028	226	-0.0149	0.1186	0.9004	0.9433
palmitoyl glycine	P-Gly	Attention	230	-0.0317	0.0604	0.5999	0.9028	226	0.0302	0.0627	0.6311	0.9433
linoleoyl leucine	L-Leu	Attention	228	0.3438	0.3852	0.3731	0.9028	224	0.4257	0.4100	0.3003	0.9433
oleoyl leucine	O-Leu	Attention	228	0.0434	0.1307	0.7403	0.9028	224	0.0296	0.1372	0.8291	0.9433
palmitoyl leucine	P-Leu	Attention	229	0.5227	0.8434	0.5360	0.9028	225	0.5831	0.9149	0.5246	0.9433
linoleoyl serine	L-Ser	Attention	230	-0.2702	0.6649	0.6849	0.9028	226	0.0606	0.6701	0.9280	0.9496
oleoyl serine	O-Ser	Attention	229	-0.2050	0.2483	0.4100	0.9028	225	-0.1065	0.2553	0.6770	0.9433
palmitoyl serine	P-Ser	Attention	230	-0.2682	0.1653	0.1061	0.7092	226	-0.1725	0.1736	0.3215	0.9433
arachidonoyl serine	A-Ser	Attention	230	-0.0446	0.0490	0.3635	0.9028	226	-0.0247	0.0489	0.6140	0.9433
linoleoyl phenylalanine	L-Phe	Attention	230	-0.0314	0.0464	0.4991	0.9028	226	-0.0315	0.0465	0.4988	0.9433
arachidonoyl leucine	A-Leu	Attention	230	0.0105	0.0461	0.8207	0.9028	226	0.0230	0.0476	0.6294	0.9433
docosahexaenoyl leucine	DH-Leu	Attention	230	0.0127	0.0520	0.8068	0.9028	226	0.0111	0.0517	0.8300	0.9433
arachidonoyl glycine	A-Gly	Attention	230	0.0004	0.0441	0.9920	0.9920	226	0.0438	0.0448	0.3297	0.9433
docosahexaenoyl glycine	DH-Gly	Attention	230	0.0217	0.0495	0.6612	0.9028	226	0.0381	0.0492	0.4403	0.9433
stearidonoyl glycine	S-Gly	Attention	230	-0.0182	0.0451	0.6869	0.9028	226	-0.0124	0.0451	0.7837	0.9433

© 2024. Vered S. et al. Published online at http://www.aginganddisease.org/EN/10.14336/AD.2024.1427

linoleoyl valine	L-Val	Attention	230	0.0108	0.0443	0.8067	0.9028	226	0.0244	0.0454	0.5912	0.9433
oleoyl valine	O-Val	Attention	230	0.0403	0.0483	0.4043	0.9028	226	0.0528	0.0481	0.2733	0.9433
arachidonoyl glycerol 2&1	2-AG	Attention	230	0.0054	0.0444	0.9037	0.9467	226	0.0261	0.0446	0.5585	0.9433
arachidonic acid	AA	Executive function	224	-0.0008	0.0003	0.0054	0.0264	220	-0.0007	0.0003	0.0150	0.0943
docosahexaenoic acid	DHA	Executive function	223	-0.0004	0.0003	0.1938	0.3553	219	-0.0003	0.0003	0.3240	0.7128
eicosapentaenoic acid	EPA	Executive function	221	-0.0003	0.0006	0.6424	0.7438	217	0.0000	0.0006	0.9605	0.9902
linoleic acid	LA	Executive function	224	-0.0003	0.0001	<.0001	0.0022	220	-0.0002	0.0001	0.0023	0.0363
linolenic acid	LnA	Executive function	224	-0.0006	0.0001	<.0001	0.0022	220	-0.0005	0.0001	0.0005	0.0220
oleic acid	OA	Executive function	224	-0.0002	0.0000	0.0002	0.0029	220	-0.0002	0.0001	0.0033	0.0363
palmitic acid	PA	Executive function	224	-0.0003	0.0001	0.0005	0.0053	220	-0.0003	0.0001	0.0078	0.0572
stearic acid	SA	Executive function	224	-0.0001	0.0001	0.3898	0.5814	220	0.0000	0.0001	0.9133	0.9902
arachidonoyl ethanolamide	AEA	Executive function	223	-0.5789	0.3889	0.1380	0.2760	219	-0.2688	0.3922	0.4940	0.8502
docosahexaenoyl ethanolamide	DHEA	Executive function	224	-0.0898	0.2340	0.7016	0.7915	220	0.0795	0.2416	0.7423	0.9606
linoleoyl ethanolamide	LEA	Executive function	223	-0.3826	0.2243	0.0894	0.2185	219	-0.0973	0.2327	0.6762	0.9606
oleoyl ethanolamide	OEA	Executive function	222	-0.1964	0.1312	0.1359	0.2760	218	-0.0758	0.1383	0.5844	0.8931
palmitoyl ethanolamide	PEA	Executive function	223	-0.2029	0.0942	0.0324	0.1103	219	-0.0912	0.0979	0.3526	0.7388
stearoyl ethanolamide	SEA	Executive function	223	-0.1621	0.1491	0.2780	0.4893	219	0.0012	0.1505	0.9937	0.9937
docosahexaenoyl glycerol 2&1	2-DHG	Executive function	221	0.0382	0.0737	0.6051	0.7321	217	0.0181	0.0741	0.8074	0.9806
linolenoyl glycerol 2&1	2-LnG	Executive function	222	-0.0504	0.0888	0.5708	0.7193	219	-0.0333	0.0870	0.7026	0.9606
oleoyl glycerol 2&1	2-OG	Executive function	219	-0.0169	0.0186	0.3627	0.5814	216	-0.0106	0.0182	0.5605	0.8931
palmitoyl glycerol 2&1	2-PG	Executive function	224	0.0001	0.0024	0.9629	0.9629	220	0.0001	0.0024	0.9596	0.9902
steareoyl glycerol 2&1	2-SG	Executive function	222	-0.0002	0.0007	0.8007	0.8442	218	0.0000	0.0007	0.9516	0.9902
linoleoyl alanine	L-Ala	Executive function	224	-3.3434	1.3473	0.0138	0.0552	220	-2.3511	1.4004	0.0947	0.3578
oleoyl alanine	O-Ala	Executive function	224	-0.8312	0.2443	0.0008	0.0059	220	-0.7440	0.2495	0.0032	0.0363

© 2024. Vered S. et al. Published online at http://www.aginganddisease.org/EN/10.14336/AD.2024.1427

palmitoyl alanine	P-Ala	Executive function	224	-2.0827	0.6009	0.0006	0.0053	220	-1.7517	0.6201	0.0052	0.0458
linolenoyl amide	Ln-Am	Executive function	221	-0.0803	0.0373	0.0326	0.1103	217	-0.0674	0.0368	0.0686	0.3559
linoleoyl amide	L-Am	Executive function	222	-0.0026	0.0040	0.5259	0.7012	218	-0.0014	0.0039	0.7252	0.9606
palmitoyl amide	P-Am	Executive function	222	-0.0049	0.0025	0.0465	0.1364	218	-0.0034	0.0024	0.1580	0.4584
linoleoyl glycine	L-Gly	Executive function	223	-0.4952	0.2388	0.0393	0.1235	219	-0.2783	0.2440	0.2553	0.5912
oleoyl glycine	O-Gly	Executive function	224	-0.3187	0.1083	0.0036	0.0226	220	-0.1876	0.1150	0.1042	0.3578
palmitoyl glycine	P-Gly	Executive function	224	-0.1679	0.0589	0.0048	0.0264	220	-0.1066	0.0613	0.0833	0.3578
linoleoyl leucine	L-Leu	Executive function	222	0.0951	0.3862	0.8058	0.8442	218	-0.0198	0.4119	0.9617	0.9902
oleoyl leucine	O-Leu	Executive function	222	0.0321	0.1288	0.8034	0.8442	218	-0.0473	0.1347	0.7257	0.9606
palmitoyl leucine	P-Leu	Executive function	223	0.7284	0.8316	0.3820	0.5814	219	0.2219	0.8966	0.8047	0.9806
linoleoyl serine	L-Ser	Executive function	224	-1.0299	0.6532	0.1163	0.2693	220	-0.7522	0.6541	0.2515	0.5912
oleoyl serine	O-Ser	Executive function	223	-0.6315	0.2465	0.0111	0.0488	219	-0.4565	0.2532	0.0728	0.3559
palmitoyl serine	P-Ser	Executive function	224	-0.2861	0.1666	0.0873	0.2185	220	-0.2806	0.1727	0.1057	0.3578
arachidonoyl serine	A-Ser	Executive function	224	0.0409	0.0482	0.3964	0.5814	220	0.0661	0.0476	0.1667	0.4584
linoleoyl phenylalanine	L-Phe	Executive function	224	0.0406	0.0462	0.3800	0.5814	220	0.0311	0.0461	0.5010	0.8502
arachidonoyl leucine	A-Leu	Executive function	224	-0.0040	0.0456	0.9307	0.9523	220	-0.0104	0.0469	0.8246	0.9806
docosahexaenoyl leucine	DH-Leu	Executive function	224	0.0801	0.0520	0.1246	0.2741	220	0.0774	0.0512	0.1324	0.4161
arachidonoyl glycine	A-Gly	Executive function	224	-0.0779	0.0433	0.0734	0.2019	220	-0.0386	0.0440	0.3814	0.7499
docosahexaenoyl glycine	DH-Gly	Executive function	224	0.0280	0.0495	0.5722	0.7193	220	0.0420	0.0490	0.3920	0.7499
stearidonoyl glycine	S-Gly	Executive function	224	-0.0324	0.0450	0.4732	0.6507	220	-0.0300	0.0447	0.5024	0.8502
linoleoyl valine	L-Val	Executive function	224	0.0621	0.0439	0.1589	0.3040	220	0.0594	0.0448	0.1863	0.4822
oleoyl valine	O-Val	Executive function	224	-0.0344	0.0475	0.4700	0.6507	220	-0.0256	0.0472	0.5886	0.8931
arachidonoyl glycerol 2&1	2-AG	Executive function	224	-0.0222	0.0441	0.6156	0.7321	220	0.0018	0.0441	0.9677	0.9902

© 2024. Vered S. et al. Published online at http://www.aginganddisease.org/EN/10.14336/AD.2024.1427

SUPPLEMENTARY DATA

Cognitive function: Verbal memory = Logical Memory-Delayed Recall test; Visual memory = Visual Reproductions-Delayed Recall test; Abstract reasoning = Similarities test; Perceptual organization = Hooper Visual Organization Test; Attention = Trail-making Test A; Executive function = Trail-making Test B minus A. Models adjusted for age, age squared, sex, education, apolipoprotein ε4 genotype, obesity and time between blood draw and cognitive assessment.

Supplementary Table 4. Main sample characteristics by sex and apolipoprotein ε4 genotype.

Variables	Women N=142 (60%)	Men N=95 (40%)	No Apolipoprotein ε4 genotype N=186 (80%)	Apolipoprotein ε4 genotype N=46 (20%)
Age, y	73.6±6.6	72.8±5.5	73.7±6.2	72.4±5.9
Sex (men)	-	-	74 (39.8)	20 (43.5)
Education (college)	93 (65.5)	72 (75.8)	129 (69.3)	31 (67.4)
Apolipoprotein ε4 genotype	26 (18.8)	20 (21.3)	-	-
Obesity	40 (28.2)	24 (25.3)	54 (29.0)	10 (21.7)
Time between blood draw and cognitive assessment, y	1.6 ±1.0	1.7 ±1.0	1.6 ±1.0	1.8 ±1.0
**Cognitive function**				
Verbal memory	11.7 ±4.0	10.7 ±3.7	11.3 ±3.9	10.1 ±3.8
Visual memory	6.4 ±3.0	6.7 ±3.0	6.6 ±2.9	6.2 ±3.2
Abstract reasoning	16.3 ±4.1	16.6 ±3.3	16.3 ±3.9	16.6 ±3.2
Perceptual organization	25.5 [23.5-27.0]	24.5 [22.5-26.5]	25.0 [23.0-27.0]	24.8 [23.3-27.5]
Attention	0.6 [0.5-0.7]	0.6 [0.5-0.7]	0.6 [0.5-0.7]	0.6 [0.5-0.7]
Executive function	0.9 [0.6-1.5]	0.9 [0.6-1.4]	0.9 [0.6-1.4]	1.0 [0.7-1.7]

Cognitive function: Verbal memory = Logical Memory-Delayed Recall test; Visual memory = Visual Reproductions-Delayed Recall test; Abstract reasoning = Similarities test; Perceptual organization = Hooper Visual Organization Test; Attention = Trail-making Test A; Executive function = Trail-making Test B minus A.
Continuous traits values are reported as mean ±SD or median [IQR] and dichotomous traits values are reported as number (percent).

Supplementary Table 5. Interactions between levels of each of the 44 eCBs compounds and sex / apolipoprotein ε4 genotype, in relation to cognitive function in the total sample.

Endocannabinoids	Abbreviation	Outcomes	Interaction with sex				Interaction with apolipoprotein ε4 genotype			
			N	Parameter Estimate	Standard Error	P value	N	Parameter Estimate	Standard Error	P value
arachidonic acid	AA	Verbal memory	226	0.0135	0.0071	0.0580	226	-0.0013	0.0091	0.8885
docosahexaenoic acid	DHA	Verbal memory	226	0.0065	0.0075	0.3874	226	0.0003	0.0091	0.9700
eicosapentaenoic acid	EPA	Verbal memory	224	0.0228	0.0160	0.1555	224	0.0035	0.0199	0.8619
linoleic acid	LA	Verbal memory	226	0.0035	0.0020	0.0767	226	-0.0011	0.0026	0.6772
linolenic acid	LnA	Verbal memory	226	0.0075	0.0039	0.0593	226	-0.0022	0.0055	0.6920
oleic acid	OA	Verbal memory	226	0.0021	0.0013	0.1241	226	-0.0006	0.0016	0.7326

© 2024. Vered S. et al. Published online at http://www.aginganddisease.org/EN/10.14336/AD.2024.1427

SUPPLEMENTARY DATA

palmitic acid	PA	Verbal memory	226	0.0029	0.0026	0.2625	226	-0.0020	0.0033	0.5349
stearic acid	SA	Verbal memory	226	-0.0023	0.0024	0.3292	226	-0.0030	0.0031	0.3303
arachidonoyl ethanolamide	AEA	Verbal memory	225	-3.2741	9.6233	0.7340	225	-5.1354	11.0791	0.6435
docosahexaenoyl ethanolamide	DHEA	Verbal memory	226	-1.0204	5.8632	0.8620	226	3.3584	7.5263	0.6559
linoleoyl ethanolamide	LEA	Verbal memory	225	2.1790	6.0848	0.7206	225	-2.2894	6.6425	0.7307
oleoyl ethanolamide	OEA	Verbal memory	225	-1.8760	3.9566	0.6359	225	0.4755	3.8964	0.9030
palmitoyl ethanolamide	PEA	Verbal memory	226	-3.2807	2.4881	0.1887	226	-2.5726	2.8100	0.3609
stearoyl ethanolamide	SEA	Verbal memory	226	-6.5649	3.8874	0.0927	226	3.7518	4.3779	0.3924
docosahexaenoyl glycerol 2&1	2-DHG	Verbal memory	223	0.7200	2.0259	0.7226	223	0.3347	2.3494	0.8869
linolenoyl glycerol 2&1	2-LnG	Verbal memory	225	3.4141	2.2016	0.1224	225	0.0601	2.7110	0.9823
oleoyl glycerol 2&1	2-OG	Verbal memory	222	0.2704	0.4421	0.5414	222	-0.1058	0.5271	0.8411
palmitoyl glycerol 2&1	2-PG	Verbal memory	226	0.0450	0.0594	0.4494	226	0.1086	0.0729	0.1377
steareoyl glycerol 2&1	2-SG	Verbal memory	224	0.0197	0.0164	0.2329	224	0.0218	0.0196	0.2664
linoleoyl alanine	L-Ala	Verbal memory	225	50.5517	32.9714	0.1267	225	7.9288	45.8069	0.8627
oleoyl alanine	O-Ala	Verbal memory	225	3.4660	6.2584	0.5803	225	-0.7122	10.4965	0.9460
palmitoyl alanine	P-Ala	Verbal memory	225	4.7857	15.4322	0.7568	225	-3.5887	24.0632	0.8816
linolenoyl amide	Ln-Am	Verbal memory	223	1.6366	0.9324	0.0807	223	-1.8800	1.2947	0.1480
linoleoyl amide	L-Am	Verbal memory	224	0.1070	0.0994	0.2825	224	-0.1506	0.1126	0.1823
palmitoyl amide	P-Am	Verbal memory	224	0.0484	0.0616	0.4330	224	-0.0271	0.0735	0.7130
linoleoyl glycine	L-Gly	Verbal memory	225	4.7425	5.8242	0.4164	225	-5.6272	7.1416	0.4316
oleoyl glycine	O-Gly	Verbal memory	226	1.3537	2.9793	0.6500	226	0.3261	3.2829	0.9210
palmitoyl glycine	P-Gly	Verbal memory	226	-0.1441	1.4907	0.9231	226	-0.2848	1.8852	0.8801
linoleoyl leucine	L-Leu	Verbal memory	224	14.9567	9.8437	0.1301	224	-0.3837	10.9140	0.9720
oleoyl leucine	O-Leu	Verbal memory	224	5.2097	3.3377	0.1200	224	-0.1088	3.5581	0.9756
palmitoyl leucine	P-Leu	Verbal memory	225	-0.5662	21.4674	0.9790	225	10.4021	26.6489	0.6967
linoleoyl serine	L-Ser	Verbal memory	226	24.5790	15.5546	0.1155	226	-1.7734	23.2761	0.9393
oleoyl serine	O-Ser	Verbal memory	225	3.5176	6.2795	0.5759	225	7.4768	7.5623	0.3239
palmitoyl serine	P-Ser	Verbal memory	226	-4.3811	4.1984	0.2979	226	-4.5583	5.1381	0.3760
arachidonoyl serine	A-Ser	Verbal memory	226	-0.1115	1.1710	0.9242	226	-0.1468	1.4870	0.9215
linoleoyl phenylalanine	L-Phe	Verbal memory	226	1.2761	1.1074	0.2505	226	0.4708	1.3195	0.7216
arachidonoyl leucine	A-Leu	Verbal memory	226	1.1220	1.2323	0.3636	226	3.2672	1.3502	0.0164
docosahexaenoyl leucine	DH-Leu	Verbal memory	226	-0.4909	1.2512	0.6952	226	-0.1738	1.4705	0.9060
arachidonoyl glycine	A-Gly	Verbal memory	226	0.6120	1.0643	0.5659	226	-0.9173	1.3171	0.4869
docosahexaenoyl glycine	DH-Gly	Verbal memory	226	-1.1627	1.1951	0.3317	226	0.6472	1.4027	0.6450
stearidonoyl glycine	S-Gly	Verbal memory	226	2.4417	1.0929	0.0265	226	1.2028	1.3426	0.3713
linoleoyl valine	L-Val	Verbal memory	226	1.3153	1.1203	0.2417	226	1.3137	1.3197	0.3207
oleoyl valine	O-Val	Verbal memory	226	0.6234	1.1817	0.5983	226	1.5749	1.3536	0.2459
arachidonoyl glycerol 2&1	2-AG	Verbal memory	226	0.5856	1.0644	0.5828	226	-0.0531	1.3219	0.9680
arachidonic acid	AA	Visual memory	227	0.0042	0.0054	0.4351	227	0.0003	0.0068	0.9659
docosahexaenoic acid	DHA	Visual memory	227	-0.0018	0.0057	0.7556	227	-0.0026	0.0069	0.7048
eicosapentaenoic acid	EPA	Visual memory	225	-0.0006	0.0120	0.9607	225	-0.0168	0.0149	0.2602
linoleic acid	LA	Visual memory	227	-0.0014	0.0015	0.3570	227	0.0020	0.0020	0.3152
linolenic acid	LnA	Visual memory	227	-0.0019	0.0030	0.5152	227	0.0023	0.0041	0.5769
oleic acid	OA	Visual memory	227	-0.0006	0.0010	0.5668	227	0.0008	0.0012	0.4951
palmitic acid	PA	Visual memory	227	-0.0009	0.0019	0.6584	227	0.0011	0.0025	0.6481

© 2024. Vered S. et al. Published online at http://www.aginganddisease.org/EN/10.14336/AD.2024.1427

SUPPLEMENTARY DATA

stearic acid	SA	Visual memory	227	0.0004	0.0018	0.8271	227	0.0027	0.0023	0.2517
arachidonoyl ethanolamide	AEA	Visual memory	226	-7.4333	7.2116	0.3038	226	7.3277	8.3403	0.3806
docosahexaenoyl ethanolamide	DHEA	Visual memory	227	-4.9498	4.3444	0.2558	227	1.3619	5.6577	0.8100
linoleoyl ethanolamide	LEA	Visual memory	226	-10.3366	4.2344	0.0154	226	8.9985	4.9427	0.0701
oleoyl ethanolamide	OEA	Visual memory	225	-2.6229	2.9514	0.3752	225	3.1528	2.9112	0.2800
palmitoyl ethanolamide	PEA	Visual memory	226	-4.1427	1.8383	0.0252	226	-0.1583	2.1122	0.9403
stearoyl ethanolamide	SEA	Visual memory	226	-4.5707	2.9063	0.1172	226	4.2395	3.3066	0.2012
docosahexaenoyl glycerol 2&1	2-DHG	Visual memory	224	-1.2121	1.5158	0.4248	224	0.1295	1.7648	0.9416
linolenoyl glycerol 2&1	2-LnG	Visual memory	226	0.5194	1.6709	0.7562	226	1.3757	2.0754	0.5081
oleoyl glycerol 2&1	2-OG	Visual memory	223	-0.1973	0.3296	0.5501	223	-0.2492	0.3938	0.5275
palmitoyl glycerol 2&1	2-PG	Visual memory	227	0.0110	0.0446	0.8049	227	0.0986	0.0547	0.0729
steareoyl glycerol 2&1	2-SG	Visual memory	225	-0.0039	0.0124	0.7558	225	0.0274	0.0147	0.0628
linoleoyl alanine	L-Ala	Visual memory	226	-21.8744	24.6995	0.3768	226	-6.1608	34.4724	0.8583
oleoyl alanine	O-Ala	Visual memory	226	-2.8623	4.7072	0.5438	226	-1.9348	7.9423	0.8078
palmitoyl alanine	P-Ala	Visual memory	226	-15.3441	11.4908	0.1832	226	-17.3884	18.0790	0.3372
linolenoyl amide	Ln-Am	Visual memory	224	1.3957	0.6799	0.0413	224	-0.1137	0.9921	0.9089
linoleoyl amide	L-Am	Visual memory	225	0.1057	0.0688	0.1259	225	-0.0726	0.0870	0.4051
palmitoyl amide	P-Am	Visual memory	225	0.0005	0.0440	0.9910	225	-0.0510	0.0551	0.3556
linoleoyl glycine	L-Gly	Visual memory	226	-4.6371	4.3839	0.2913	226	8.3756	5.3776	0.1208
oleoyl glycine	O-Gly	Visual memory	227	-2.1022	2.2330	0.3475	227	5.0211	2.4522	0.0418
palmitoyl glycine	P-Gly	Visual memory	227	-0.1412	1.1340	0.9010	227	2.8092	1.4230	0.0496
linoleoyl leucine	L-Leu	Visual memory	225	8.6166	7.4360	0.2478	225	0.2903	8.2890	0.9721
oleoyl leucine	O-Leu	Visual memory	225	1.3928	2.5489	0.5853	225	-3.3328	2.6927	0.2172
palmitoyl leucine	P-Leu	Visual memory	226	-18.5110	16.0893	0.2512	226	-21.2381	20.1714	0.2936
linoleoyl serine	L-Ser	Visual memory	227	1.8550	11.7681	0.8749	227	4.2887	18.2619	0.8146
oleoyl serine	O-Ser	Visual memory	226	0.6789	4.7493	0.8865	226	8.1943	6.0207	0.1749
palmitoyl serine	P-Ser	Visual memory	227	-1.1237	3.1451	0.7212	227	-1.9812	4.0143	0.6221
arachidonoyl serine	A-Ser	Visual memory	227	0.1276	0.8842	0.8854	227	0.4806	1.1204	0.6684
linoleoyl phenylalanine	L-Phe	Visual memory	227	0.1359	0.8361	0.8710	227	0.0606	1.0054	0.9520
arachidonoyl leucine	A-Leu	Visual memory	227	1.7245	0.9112	0.0597	227	0.0163	1.0344	0.9874
docosahexaenoyl leucine	DH-Leu	Visual memory	227	-0.0087	0.9439	0.9926	227	-1.7976	1.1294	0.1129
arachidonoyl glycine	A-Gly	Visual memory	227	-0.2854	0.7985	0.7211	227	0.0735	0.9957	0.9412
docosahexaenoyl glycine	DH-Gly	Visual memory	227	-0.4692	0.8956	0.6009	227	0.3114	1.0737	0.7721
stearidonoyl glycine	S-Gly	Visual memory	227	-0.5421	0.8161	0.5072	227	-0.0209	1.0160	0.9836
linoleoyl valine	L-Val	Visual memory	227	0.5095	0.8343	0.5421	227	-0.0254	0.9981	0.9797
oleoyl valine	O-Val	Visual memory	227	-0.1463	0.8798	0.8681	227	0.5993	1.0224	0.5584
arachidonoyl glycerol 2&1	2-AG	Visual memory	227	0.0588	0.7952	0.9411	227	-0.2917	1.0006	0.7709
arachidonic acid	AA	Abstract reasoning	230	0.0085	0.0061	0.1658	230	-0.0003	0.0079	0.9736
docosahexaenoic acid	DHA	Abstract reasoning	229	0.0105	0.0068	0.1245	229	-0.0007	0.0083	0.9359
eicosapentaenoic acid	EPA	Abstract reasoning	227	0.0197	0.0144	0.1730	227	-0.0036	0.0180	0.8425
linoleic acid	LA	Abstract reasoning	230	0.0037	0.0017	0.0339	230	0.0038	0.0023	0.1040
linolenic acid	LnA	Abstract reasoning	230	0.0090	0.0035	0.0103	230	0.0054	0.0050	0.2773
oleic acid	OA	Abstract reasoning	230	0.0020	0.0012	0.0930	230	0.0021	0.0015	0.1585
palmitic acid	PA	Abstract reasoning	230	0.0056	0.0022	0.0106	230	0.0028	0.0028	0.3223
stearic acid	SA	Abstract reasoning	230	0.0026	0.0021	0.2144	230	0.0006	0.0028	0.8341

© 2024. Vered S. et al. Published online at http://www.aginganddisease.org/EN/10.14336/AD.2024.1427

arachidonoyl ethanolamide	AEA	Abstract reasoning	229	1.9336	8.6049	0.8224	229	6.3091	9.8438	0.5222
docosahexaenoyl ethanolamide	DHEA	Abstract reasoning	230	5.5808	5.2105	0.2853	230	-1.0005	6.7969	0.8831
linoleoyl ethanolamide	LEA	Abstract reasoning	229	10.4372	5.0885	0.0414	229	3.5869	5.9513	0.5473
oleoyl ethanolamide	OEA	Abstract reasoning	228	2.9356	3.5025	0.4029	228	2.4471	3.5195	0.4876
palmitoyl ethanolamide	PEA	Abstract reasoning	229	-0.9729	2.1893	0.6572	229	2.8519	2.4999	0.2552
stearoyl ethanolamide	SEA	Abstract reasoning	229	-3.5990	3.4936	0.3041	229	5.5916	3.9194	0.1551
docosahexaenoyl glycerol 2&1	2-DHG	Abstract reasoning	227	0.8252	1.7670	0.6410	227	0.4635	2.1192	0.8271
linolenoyl glycerol 2&1	2-LnG	Abstract reasoning	229	3.5760	1.9807	0.0724	229	-1.2373	2.4331	0.6116
oleoyl glycerol 2&1	2-OG	Abstract reasoning	226	-0.1003	0.3974	0.8010	226	-0.8336	0.4717	0.0786
palmitoyl glycerol 2&1	2-PG	Abstract reasoning	230	0.0169	0.0529	0.7491	230	0.0616	0.0627	0.3264
steareoyl glycerol 2&1	2-SG	Abstract reasoning	228	0.0139	0.0144	0.3374	228	0.0158	0.0161	0.3274
linoleoyl alanine	L-Ala	Abstract reasoning	229	88.0400	29.2309	0.0029	229	56.0667	40.8481	0.1713
oleoyl alanine	O-Ala	Abstract reasoning	229	13.6435	5.4026	0.0123	229	10.2550	8.7680	0.2434
palmitoyl alanine	P-Ala	Abstract reasoning	229	29.4693	13.4217	0.0292	229	18.5069	20.5173	0.3680
linolenoyl amide	Ln-Am	Abstract reasoning	227	0.1946	0.8253	0.8139	227	-0.2242	1.1882	0.8505
linoleoyl amide	L-Am	Abstract reasoning	228	-0.0844	0.0828	0.3089	228	-0.0938	0.1025	0.3609
palmitoyl amide	P-Am	Abstract reasoning	228	-0.1225	0.0531	0.0219	228	-0.0350	0.0660	0.5966
linoleoyl glycine	L-Gly	Abstract reasoning	229	3.6179	5.2645	0.4927	229	6.2256	6.4585	0.3361
oleoyl glycine	O-Gly	Abstract reasoning	230	1.0041	2.6605	0.7062	230	3.3993	2.9506	0.2505
palmitoyl glycine	P-Gly	Abstract reasoning	230	1.6089	1.3483	0.2341	230	1.5939	1.6691	0.3406
linoleoyl leucine	L-Leu	Abstract reasoning	228	20.8566	8.7971	0.0186	228	10.4601	9.7327	0.2837
oleoyl leucine	O-Leu	Abstract reasoning	228	3.0193	3.0056	0.3162	228	-0.1262	3.1758	0.9683
palmitoyl leucine	P-Leu	Abstract reasoning	229	35.7917	19.0264	0.0613	229	14.4065	23.6607	0.5432
linoleoyl serine	L-Ser	Abstract reasoning	230	22.9506	13.9814	0.1021	230	48.2162	20.7192	0.0209
oleoyl serine	O-Ser	Abstract reasoning	229	8.3343	5.4432	0.1272	229	9.5516	6.4838	0.1421
palmitoyl serine	P-Ser	Abstract reasoning	230	4.7026	3.6983	0.2049	230	8.1171	4.4937	0.0722
arachidonoyl serine	A-Ser	Abstract reasoning	230	0.8741	1.0432	0.4030	230	1.6116	1.3035	0.2176
linoleoyl phenylalanine	L-Phe	Abstract reasoning	230	2.0457	0.9809	0.0382	230	0.1090	1.1749	0.9261
arachidonoyl leucine	A-Leu	Abstract reasoning	230	2.6437	1.0741	0.0146	230	0.6314	1.2337	0.6093
docosahexaenoyl leucine	DH-Leu	Abstract reasoning	230	1.1242	1.1198	0.3165	230	0.2089	1.3303	0.8754
arachidonoyl glycine	A-Gly	Abstract reasoning	230	-0.1174	0.9644	0.9032	230	1.7368	1.1795	0.1423
docosahexaenoyl glycine	DH-Gly	Abstract reasoning	230	-0.4792	1.0596	0.6515	230	-0.0829	1.2561	0.9475
stearidonoyl glycine	S-Gly	Abstract reasoning	230	1.3971	0.9640	0.1487	230	-0.4714	1.2013	0.6951
linoleoyl valine	L-Val	Abstract reasoning	230	2.0597	0.9965	0.0399	230	1.0261	1.1771	0.3843
oleoyl valine	O-Val	Abstract reasoning	230	3.0740	1.0304	0.0032	230	0.5341	1.2202	0.6620
arachidonoyl glycerol 2&1	2-AG	Abstract reasoning	230	0.0500	0.9536	0.9583	230	0.8595	1.1919	0.4716
arachidonic acid	AA	Perceptual organization	216	0.0004	0.0008	0.6318	216	0.0007	0.0011	0.4882
docosahexaenoic acid	DHA	Perceptual organization	215	0.0001	0.0010	0.8805	215	0.0004	0.0012	0.7028
eicosapentaenoic acid	EPA	Perceptual organization	213	0.0002	0.0021	0.9404	213	0.0008	0.0026	0.7583
linoleic acid	LA	Perceptual organization	216	-0.0002	0.0002	0.4696	216	0.0006	0.0003	0.0425
linolenic acid	LnA	Perceptual organization	216	-0.0003	0.0005	0.5356	216	0.0017	0.0007	0.0094
oleic acid	OA	Perceptual organization	216	-0.0001	0.0002	0.6571	216	0.0004	0.0002	0.0585
palmitic acid	PA	Perceptual organization	216	-0.0001	0.0003	0.6182	216	0.0005	0.0004	0.1777
stearic acid	SA	Perceptual organization	216	-0.0001	0.0003	0.8457	216	0.0001	0.0004	0.7256
arachidonoyl ethanolamide	AEA	Perceptual organization	215	-0.8131	1.1451	0.4785	215	1.3246	1.3043	0.3110

© 2024. Vered S. et al. Published online at http://www.aginganddisease.org/EN/10.14336/AD.2024.1427

SUPPLEMENTARY DATA

docosahexaenoyl ethanolamide	DHEA	Perceptual organization	216	-0.2948	0.7241	0.6844	216	0.1639	0.9759	0.8668
linoleoyl ethanolamide	LEA	Perceptual organization	215	-0.6850	0.7354	0.3527	215	1.2680	0.7951	0.1123
oleoyl ethanolamide	OEA	Perceptual organization	215	0.0496	0.4728	0.9166	215	0.7209	0.4739	0.1297
palmitoyl ethanolamide	PEA	Perceptual organization	216	-0.3296	0.2959	0.2665	216	0.1788	0.3426	0.6024
stearoyl ethanolamide	SEA	Perceptual organization	216	-0.6693	0.5019	0.1838	216	0.3576	0.5425	0.5105
docosahexaenoyl glycerol 2&1	2-DHG	Perceptual organization	214	-0.0518	0.2371	0.8271	214	-0.2418	0.2820	0.3923
linolenoyl glycerol 2&1	2-LnG	Perceptual organization	215	0.0346	0.2729	0.8993	215	-0.3471	0.3400	0.3085
oleoyl glycerol 2&1	2-OG	Perceptual organization	212	0.0079	0.0543	0.8840	212	-0.1448	0.0644	0.0256
palmitoyl glycerol 2&1	2-PG	Perceptual organization	216	0.0078	0.0071	0.2747	216	-0.0049	0.0084	0.5590
steareoyl glycerol 2&1	2-SG	Perceptual organization	214	0.0010	0.0019	0.6052	214	-0.0009	0.0022	0.6787
linoleoyl alanine	L-Ala	Perceptual organization	215	-1.5871	4.1346	0.7015	215	-2.1615	5.5296	0.6963
oleoyl alanine	O-Ala	Perceptual organization	215	-0.2395	0.7600	0.7530	215	0.4582	1.1831	0.6990
palmitoyl alanine	P-Ala	Perceptual organization	215	-1.2878	1.8649	0.4906	215	0.1819	2.7879	0.9480
linolenoyl amide	Ln-Am	Perceptual organization	213	0.0094	0.1229	0.9392	213	-0.1640	0.1620	0.3125
linoleoyl amide	L-Am	Perceptual organization	214	-0.0028	0.0113	0.8022	214	-0.0151	0.0138	0.2757
palmitoyl amide	P-Am	Perceptual organization	214	-0.0041	0.0072	0.5704	214	-0.0044	0.0089	0.6238
linoleoyl glycine	L-Gly	Perceptual organization	215	-1.5697	0.7107	0.0283	215	1.7261	0.8658	0.0475
oleoyl glycine	O-Gly	Perceptual organization	216	-0.6893	0.3578	0.0554	216	0.8552	0.3985	0.0330
palmitoyl glycine	P-Gly	Perceptual organization	216	-0.2029	0.1810	0.2635	216	0.3598	0.2231	0.1082
linoleoyl leucine	L-Leu	Perceptual organization	214	0.8385	1.2481	0.5024	214	-0.3084	1.3161	0.8150
oleoyl leucine	O-Leu	Perceptual organization	214	0.0177	0.4152	0.9661	214	-0.3043	0.4266	0.4764
palmitoyl leucine	P-Leu	Perceptual organization	215	1.2925	2.6032	0.6201	215	-1.8047	3.1895	0.5721
linoleoyl serine	L-Ser	Perceptual organization	216	-2.5428	1.9224	0.1874	216	3.5409	2.9595	0.2329
oleoyl serine	O-Ser	Perceptual organization	215	0.1951	0.7607	0.7978	215	0.8466	0.9555	0.3766
palmitoyl serine	P-Ser	Perceptual organization	216	-0.0634	0.5164	0.9024	216	-1.1059	0.6289	0.0802
arachidonoyl serine	A-Ser	Perceptual organization	216	0.0027	0.1426	0.9847	216	-0.0377	0.1765	0.8311
linoleoyl phenylalanine	L-Phe	Perceptual organization	216	0.0287	0.1380	0.8354	216	-0.1127	0.1625	0.4889
arachidonoyl leucine	A-Leu	Perceptual organization	216	0.2786	0.1490	0.0629	216	-0.1629	0.1664	0.3287
docosahexaenoyl leucine	DH-Leu	Perceptual organization	216	0.3176	0.1548	0.0415	216	-0.1817	0.1882	0.3353
arachidonoyl glycine	A-Gly	Perceptual organization	216	-0.2539	0.1307	0.0534	216	0.1854	0.1625	0.2550
docosahexaenoyl glycine	DH-Gly	Perceptual organization	216	-0.1820	0.1458	0.2132	216	0.0884	0.1766	0.6172
stearidonoyl glycine	S-Gly	Perceptual organization	216	0.0127	0.1334	0.9240	216	0.1749	0.1641	0.2877
linoleoyl valine	L-Val	Perceptual organization	216	0.1894	0.1378	0.1709	216	-0.0367	0.1602	0.8188
oleoyl valine	O-Val	Perceptual organization	216	0.1429	0.1438	0.3216	216	0.0315	0.1666	0.8503
arachidonoyl glycerol 2&1	2-AG	Perceptual organization	216	-0.1360	0.1316	0.3028	216	-0.3099	0.1609	0.0554
arachidonic acid	AA	Attention	226	-0.0001	0.0006	0.8505	226	0.0000	0.0007	0.9979
docosahexaenoic acid	DHA	Attention	225	0.0010	0.0006	0.1242	225	0.0000	0.0008	0.9585
eicosapentaenoic acid	EPA	Attention	223	0.0020	0.0013	0.1487	223	0.0001	0.0017	0.9657
linoleic acid	LA	Attention	226	0.0001	0.0002	0.4604	226	0.0001	0.0002	0.5693
linolenic acid	LnA	Attention	226	0.0004	0.0003	0.1973	226	0.0002	0.0005	0.6805
oleic acid	OA	Attention	226	0.0001	0.0001	0.2733	226	0.0000	0.0001	0.8060
palmitic acid	PA	Attention	226	0.0002	0.0002	0.2413	226	0.0001	0.0003	0.5786
stearic acid	SA	Attention	226	0.0000	0.0002	0.9173	226	0.0000	0.0003	0.9186
arachidonoyl ethanolamide	AEA	Attention	225	0.3641	0.8155	0.6557	225	-0.0023	0.9311	0.9980
docosahexaenoyl ethanolamide	DHEA	Attention	226	0.5231	0.4843	0.2813	226	0.6041	0.6314	0.3397

© 2024. Vered S. et al. Published online at http://www.aginganddisease.org/EN/10.14336/AD.2024.1427

SUPPLEMENTARY DATA

linoleoyl ethanolamide	LEA	Attention	225	0.0626	0.4827	0.8969	225	0.4660	0.5573	0.4040
oleoyl ethanolamide	OEA	Attention	224	0.3826	0.3285	0.2455	224	0.3309	0.3300	0.3170
palmitoyl ethanolamide	PEA	Attention	225	0.2637	0.2074	0.2049	225	0.0012	0.2373	0.9961
stearoyl ethanolamide	SEA	Attention	225	0.3642	0.3320	0.2739	225	0.4501	0.3746	0.2308
docosahexaenoyl glycerol 2&1	2-DHG	Attention	223	0.1220	0.1639	0.4573	223	0.1381	0.1965	0.4829
linolenoyl glycerol 2&1	2-LnG	Attention	225	0.1808	0.1868	0.3342	225	0.1907	0.2308	0.4097
oleoyl glycerol 2&1	2-OG	Attention	222	0.0255	0.0371	0.4929	222	0.0333	0.0442	0.4515
palmitoyl glycerol 2&1	2-PG	Attention	226	-0.0068	0.0049	0.1692	226	0.0067	0.0058	0.2535
steareoyl glycerol 2&1	2-SG	Attention	224	-0.0008	0.0014	0.5497	224	0.0021	0.0015	0.1613
linoleoyl alanine	L-Ala	Attention	226	0.1113	2.7937	0.9683	226	0.1721	3.8360	0.9642
oleoyl alanine	O-Ala	Attention	226	1.0659	0.5106	0.0380	226	0.1385	0.8258	0.8669
palmitoyl alanine	P-Ala	Attention	226	2.3876	1.2693	0.0613	226	0.6348	1.9401	0.7438
linolenoyl amide	Ln-Am	Attention	223	0.0405	0.0757	0.5931	223	-0.0589	0.1086	0.5882
linoleoyl amide	L-Am	Attention	224	0.0150	0.0076	0.0497	224	-0.0070	0.0094	0.4592
palmitoyl amide	P-Am	Attention	224	0.0036	0.0051	0.4831	224	0.0034	0.0062	0.5854
linoleoyl glycine	L-Gly	Attention	225	-0.1635	0.4942	0.7412	225	0.1391	0.6067	0.8189
oleoyl glycine	O-Gly	Attention	226	0.2466	0.2493	0.3237	226	0.2008	0.2762	0.4680
palmitoyl glycine	P-Gly	Attention	226	0.0387	0.1259	0.7589	226	0.1263	0.1563	0.4202
linoleoyl leucine	L-Leu	Attention	224	0.1969	0.8405	0.8150	224	0.3013	0.9239	0.7447
oleoyl leucine	O-Leu	Attention	224	0.0211	0.2931	0.9428	224	-0.0627	0.3029	0.8361
palmitoyl leucine	P-Leu	Attention	225	-0.0811	1.8281	0.9646	225	0.9330	2.2317	0.6763
linoleoyl serine	L-Ser	Attention	226	-1.9770	1.3257	0.1374	226	0.5098	2.0421	0.8031
oleoyl serine	O-Ser	Attention	225	0.0156	0.5153	0.9758	225	0.7166	0.6357	0.2608
palmitoyl serine	P-Ser	Attention	226	0.1984	0.3512	0.5727	226	-0.2095	0.4400	0.6346
arachidonoyl serine	A-Ser	Attention	226	-0.0381	0.0989	0.7005	226	0.2770	0.1216	0.0236
linoleoyl phenylalanine	L-Phe	Attention	226	0.0073	0.0938	0.9381	226	0.1681	0.1110	0.1314
arachidonoyl leucine	A-Leu	Attention	226	-0.1235	0.1019	0.2272	226	0.1093	0.1157	0.3457
docosahexaenoyl leucine	DH-Leu	Attention	226	0.0342	0.1060	0.7474	226	-0.1895	0.1271	0.1374
arachidonoyl glycine	A-Gly	Attention	226	0.0066	0.0907	0.9419	226	-0.0452	0.1110	0.6845
docosahexaenoyl glycine	DH-Gly	Attention	226	-0.0075	0.0999	0.9399	226	-0.0042	0.1202	0.9722
stearidonoyl glycine	S-Gly	Attention	226	-0.1597	0.0909	0.0802	226	-0.0464	0.1137	0.6838
linoleoyl valine	L-Val	Attention	226	-0.0128	0.0943	0.8924	226	0.0359	0.1109	0.7465
oleoyl valine	O-Val	Attention	226	0.0464	0.0984	0.6381	226	0.1665	0.1139	0.1451
arachidonoyl glycerol 2&1	2-AG	Attention	226	0.0178	0.0895	0.8429	226	0.1797	0.1116	0.1088
arachidonic acid	AA	Executive function	220	0.0007	0.0006	0.2461	220	0.0001	0.0007	0.8563
docosahexaenoic acid	DHA	Executive function	219	0.0004	0.0006	0.5822	219	0.0002	0.0008	0.7797
eicosapentaenoic acid	EPA	Executive function	217	0.0007	0.0013	0.5860	217	0.0001	0.0016	0.9532
linoleic acid	LA	Executive function	220	0.0002	0.0002	0.1346	220	0.0004	0.0002	0.0738
linolenic acid	LnA	Executive function	220	0.0007	0.0003	0.0286	220	0.0007	0.0004	0.1276
oleic acid	OA	Executive function	220	0.0002	0.0001	0.0760	220	0.0002	0.0001	0.1580
palmitic acid	PA	Executive function	220	0.0003	0.0002	0.0905	220	0.0003	0.0003	0.2230
stearic acid	SA	Executive function	220	-0.0001	0.0002	0.6145	220	0.0001	0.0003	0.8423
arachidonoyl ethanolamide	AEA	Executive function	219	0.7876	0.7962	0.3237	219	1.6639	0.9257	0.0737
docosahexaenoyl ethanolamide	DHEA	Executive function	220	-0.0227	0.4790	0.9622	220	0.2229	0.6197	0.7194
linoleoyl ethanolamide	LEA	Executive function	219	0.5240	0.4751	0.2713	219	1.3961	0.5383	0.0102

© 2024. Vered S. et al. Published online at http://www.aginganddisease.org/EN/10.14336/AD.2024.1427

oleoyl ethanolamide	OEA	Executive function	218	0.4198	0.3136	0.1821	218	0.5094	0.3140	0.1062
palmitoyl ethanolamide	PEA	Executive function	219	-0.0289	0.2026	0.8867	219	0.2756	0.2313	0.2349
stearoyl ethanolamide	SEA	Executive function	219	0.1078	0.3252	0.7405	219	0.4987	0.3679	0.1768
docosahexaenoyl glycerol 2&1	2-DHG	Executive function	217	-0.0907	0.1624	0.5771	217	-0.2252	0.1937	0.2465
linolenoyl glycerol 2&1	2-LnG	Executive function	219	0.0140	0.1843	0.9397	219	-0.2473	0.2276	0.2784
oleoyl glycerol 2&1	2-OG	Executive function	216	-0.0062	0.0370	0.8680	216	-0.0858	0.0437	0.0510
palmitoyl glycerol 2&1	2-PG	Executive function	220	-0.0024	0.0048	0.6214	220	0.0033	0.0057	0.5683
steareoyl glycerol 2&1	2-SG	Executive function	218	0.0002	0.0013	0.8727	218	0.0013	0.0015	0.3788
linoleoyl alanine	L-Ala	Executive function	220	5.3478	2.7097	0.0497	220	3.8558	3.7151	0.3005
oleoyl alanine	O-Ala	Executive function	220	1.1415	0.4939	0.0218	220	0.3603	0.7918	0.6496
palmitoyl alanine	P-Ala	Executive function	220	1.6195	1.2383	0.1923	220	-0.6534	1.8776	0.7282
linolenoyl amide	Ln-Am	Executive function	217	0.1296	0.0759	0.0891	217	-0.0347	0.1208	0.7744
linoleoyl amide	L-Am	Executive function	218	0.0065	0.0080	0.4180	218	-0.0111	0.0116	0.3376
palmitoyl amide	P-Am	Executive function	218	-0.0014	0.0050	0.7754	218	-0.0082	0.0061	0.1757
linoleoyl glycine	L-Gly	Executive function	219	0.0764	0.4810	0.8739	219	0.8462	0.5872	0.1510
oleoyl glycine	O-Gly	Executive function	220	0.1322	0.2419	0.5851	220	0.5167	0.2650	0.0525
palmitoyl glycine	P-Gly	Executive function	220	0.0908	0.1227	0.4601	220	0.2806	0.1511	0.0648
linoleoyl leucine	L-Leu	Executive function	218	1.2362	0.8577	0.1510	218	0.4986	0.9103	0.5845
oleoyl leucine	O-Leu	Executive function	218	0.5134	0.2872	0.0753	218	-0.0189	0.2964	0.9491
palmitoyl leucine	P-Leu	Executive function	219	0.6434	1.7954	0.7204	219	0.7818	2.1939	0.7219
linoleoyl serine	L-Ser	Executive function	220	2.7252	1.2889	0.0357	220	2.3209	1.9750	0.2413
oleoyl serine	O-Ser	Executive function	219	0.8079	0.5090	0.1140	219	0.5884	0.6267	0.3488
palmitoyl serine	P-Ser	Executive function	220	0.2262	0.3495	0.5182	220	-0.2628	0.4375	0.5488
arachidonoyl serine	A-Ser	Executive function	220	-0.0394	0.0963	0.6830	220	0.0181	0.1194	0.8800
linoleoyl phenylalanine	L-Phe	Executive function	220	0.1110	0.0922	0.2299	220	0.0612	0.1093	0.5764
arachidonoyl leucine	A-Leu	Executive function	220	0.1595	0.0991	0.1090	220	0.0842	0.1152	0.4655
docosahexaenoyl leucine	DH-Leu	Executive function	220	-0.0639	0.1047	0.5419	220	-0.1793	0.1274	0.1607
arachidonoyl glycine	A-Gly	Executive function	220	0.0766	0.0888	0.3895	220	0.0148	0.1089	0.8923
docosahexaenoyl glycine	DH-Gly	Executive function	220	-0.0635	0.0988	0.5210	220	-0.0045	0.1200	0.9704
stearidonoyl glycine	S-Gly	Executive function	220	0.0655	0.0906	0.4706	220	-0.0090	0.1129	0.9366
linoleoyl valine	L-Val	Executive function	220	0.0344	0.0923	0.7098	220	0.0233	0.1084	0.8301
oleoyl valine	O-Val	Executive function	220	0.1354	0.0961	0.1603	220	0.0424	0.1124	0.7065
arachidonoyl glycerol 2&1	2-AG	Executive function	220	-0.0255	0.0882	0.7725	220	-0.1265	0.1104	0.2531

Cognitive function: Verbal memory = Logical Memory-Delayed Recall test; Visual memory = Visual Reproductions-Delayed Recall test; Abstract reasoning = Similarities test; Perceptual organization = Hooper Visual Organization Test; Attention = Trail-making Test A; Executive function = Trail-making Test B minus A. Models adjusted for age, age squared, sex, education, apolipoprotein ε4 genotype, obesity and time between blood draw and cognitive assessment.

© 2024. Vered S. et al. Published online at http://www.aginganddisease.org/EN/10.14336/AD.2024.1427

Supplementary Table 6. Significant interactions between eCBs levels and cognitive function in the various domains by sex. eCBs with at least one interaction with sex (P<0.1) are shown.

Endocannabinoids	Abbreviation	Outcomes	Women					Men				
			N	Parameter Estimate	Standard Error	standardized Parameter Estimate	P value	N	Parameter Estimate	Standard Error	standardized Parameter Estimate	P value
arachidonic acid	AA	Verbal memory	137	-0.0074	0.0045	-0.1456	0.1003	89	0.0067	0.0054	0.1320	0.2197
linoleic acid	LA	Verbal memory	137	-0.0026	0.0011	-0.2004	0.0223	89	0.0008	0.0016	0.0533	0.6161
linolenic acid	LnA	Verbal memory	137	-0.0061	0.0022	-0.2349	0.0063	89	0.0017	0.0032	0.0550	0.6069
stearoyl ethanolamide	SEA	Verbal memory	137	1.4150	2.2427	0.0566	0.5292	89	-5.1897	3.1038	-0.1775	0.0984
linolenoyl amide	Ln-Am	Verbal memory	135	-2.0119	0.5610	-0.3008	0.0005	88	-0.4387	0.7387	-0.0639	0.5543
stearidonoyl glycine	S-Gly	Verbal memory	137	-1.4431	0.7046	-0.1771	0.0426	89	0.7711	0.8357	0.0981	0.3589
linoleoyl ethanolamide	LEA	Visual memory	135	2.3622	2.5322	0.0823	0.3527	91	-6.8593	3.5464	-0.1952	0.0565
palmitoyl ethanolamide	PEA	Visual memory	136	1.2569	1.1232	0.0996	0.2652	90	-2.1624	1.4923	-0.1480	0.1511
linolenoyl amide	Ln-Am	Visual memory	134	-0.7732	0.4198	-0.1576	0.0679	90	0.5391	0.5333	0.1015	0.3151
arachidonoyl leucine	A-Leu	Visual memory	136	-0.6534	0.5319	-0.1102	0.2216	91	0.8334	0.7726	0.1135	0.2839
linoleic acid	LA	Abstract reasoning	137	-0.0016	0.0011	-0.1232	0.1212	93	0.0019	0.0013	0.1467	0.1483
linolenic acid	LnA	Abstract reasoning	137	-0.0025	0.0021	-0.0945	0.2279	93	0.0065	0.0026	0.2429	0.0158
oleic acid	OA	Abstract reasoning	137	-0.0009	0.0008	-0.0965	0.2310	93	0.0012	0.0009	0.1451	0.1579
palmitic acid	PA	Abstract reasoning	137	-0.0028	0.0014	-0.1571	0.0492	93	0.0028	0.0016	0.1764	0.0836
linoleoyl ethanolamide	LEA	Abstract reasoning	136	-0.8522	3.2048	-0.0214	0.7907	93	8.4419	3.9664	0.2191	0.0362
linolenoyl glycerol 2&1	2-LnG	Abstract reasoning	137	-2.4688	1.2295	-0.1548	0.0467	92	0.6934	1.5173	0.0480	0.6489
linoleoyl alanine	L-Ala	Abstract reasoning	136	-45.4926	22.4759	-0.1728	0.0450	93	43.6794	20.1343	0.2193	0.0328
oleoyl alanine	O-Ala	Abstract reasoning	136	-11.2408	4.0584	-0.2339	0.0064	93	3.7272	3.7322	0.1026	0.3208
palmitoyl alanine	P-Ala	Abstract reasoning	136	-28.8629	9.7380	-0.2522	0.0036	93	4.1963	9.7344	0.0448	0.6675
palmitoyl amide	P-Am	Abstract reasoning	136	0.0354	0.0344	0.0800	0.3061	92	-0.0910	0.0387	-0.2379	0.0212
linoleoyl leucine	L-Leu	Abstract reasoning	137	-8.6766	7.3574	-0.0911	0.2404	91	10.6372	5.0268	0.2153	0.0373
palmitoyl leucine	P-Leu	Abstract reasoning	137	-8.9541	14.9351	-0.0480	0.5499	92	29.2102	11.9545	0.2443	0.0166
linoleoyl phenylalanine	L-Phe	Abstract reasoning	137	-0.2124	0.7046	-0.0235	0.7636	93	1.8360	0.6606	0.2746	0.0067
arachidonoyl leucine	A-Leu	Abstract reasoning	137	-0.3075	0.6740	-0.0377	0.6490	93	2.4969	0.8163	0.3150	0.0030
linoleoyl valine	L-Val	Abstract reasoning	137	-0.3861	0.7209	-0.0441	0.5931	93	1.6519	0.6708	0.2518	0.0158
oleoyl valine	O-Val	Abstract reasoning	137	-0.8092	0.7198	-0.0911	0.2630	93	2.2501	0.7116	0.3063	0.0022
linoleoyl glycine	L-Gly	Perceptual organization	127	0.8897	0.5097	0.1540	0.0835	88	-0.7210	0.5180	-0.1496	0.1678
oleoyl glycine	O-Gly	Perceptual organization	128	0.2715	0.2262	0.1120	0.2324	88	-0.4295	0.2780	-0.1632	0.1263
arachidonoyl leucine	A-Leu	Perceptual organization	128	0.0221	0.0922	0.0222	0.8112	88	0.2865	0.1206	0.2591	0.0199

© 2024. Vered S. et al. Published online at http://www.aginganddisease.org/EN/10.14336/AD.2024.1427

docosahexaenoyl leucine	DH-Leu	Perceptual organization	128	-0.0995	0.1042	-0.0839	0.3415	88	0.2253	0.1141	0.2079	0.0517
arachidonoyl glycine	A-Gly	Perceptual organization	128	0.0990	0.0915	0.0986	0.2811	88	-0.1729	0.0947	-0.1931	0.0714
oleoyl alanine	O-Ala	Attention	134	-0.5222	0.3910	-0.1270	0.1841	92	0.6575	0.3426	0.1976	0.0584
palmitoyl alanine	P-Ala	Attention	134	-0.8252	0.9446	-0.0845	0.3840	92	1.7279	0.8870	0.2014	0.0548
linoleoyl amide	L-Am	Attention	133	-0.0189	0.0049	-0.3126	0.0002	91	-0.0017	0.0057	-0.0315	0.7668
stearidonoyl glycine	S-Gly	Attention	134	0.0485	0.0620	0.0679	0.4352	92	-0.1016	0.0649	-0.1604	0.1211
linolenic acid	LnA	Executive function	130	-0.0007	0.0002	-0.3111	0.0002	90	-0.0001	0.0002	-0.0241	0.8214
oleic acid	OA	Executive function	130	-0.0002	0.0001	-0.2631	0.0026	90	0.0000	0.0001	-0.0470	0.6627
palmitic acid	PA	Executive function	130	-0.0004	0.0001	-0.2406	0.0061	90	0.0000	0.0001	-0.0329	0.7599
linoleoyl alanine	L-Ala	Executive function	130	-5.1026	2.0930	-0.2269	0.0162	90	0.7589	1.8433	0.0445	0.6816
oleoyl alanine	O-Ala	Executive function	130	-1.3625	0.3729	-0.3319	0.0004	90	-0.0785	0.3383	-0.0251	0.8171
linolenoyl amide	Ln-Am	Executive function	128	-0.1186	0.0514	-0.1983	0.0227	89	0.0000	0.0534	-0.0001	0.9996
oleoyl leucine	O-Leu	Executive function	130	-0.3709	0.2519	-0.1271	0.1435	88	0.1594	0.1504	0.1151	0.2925
linoleoyl serine	L-Ser	Executive function	130	-1.9306	0.9270	-0.1773	0.0394	90	0.7469	0.9112	0.0911	0.4148

Cognitive function: Verbal memory = Logical Memory-Delayed Recall test; Visual memory = Visual Reproductions-Delayed Recall test; Abstract reasoning = Similarities test; Perceptual organization = Hooper Visual Organization Test; Attention = Trail-making Test A; Executive function = Trail-making Test B minus A. Models adjusted for age, age squared, education, apolipoprotein ε4 genotype, obesity and time between blood draw and cognitive assessment.

Supplementary Table 7. Significant interactions between eCBs levels and cognitive function in the various domains by apolipoprotein ε4 genotype. eCBs with at least one interaction with apolipoprotein ε4 genotype ($P<0.1$) are shown.

**Endocannabinoids**	**Abbreviation**	**Outcomes**	**no apolipoprotein ε4 genotype**					**with apolipoprotein ε4 genotype**				
			**N**	**Parameter Estimate**	**Standard Error**	**standardized Parameter Estimate**	**P value**	**N**	**Parameter Estimate**	**Standard Error**	**standardized Parameter Estimate**	**P value**
Arachidonoyl leucine	A-Leu	Verbal memory	181	-1.4198	0.6349	-0.1737	0.0266	45	3.0210	1.2486	0.3734	0.0206
linoleoyl ethanolamide	LEA	Visual memory	182	-2.3316	2.3387	-0.0767	0.3202	44	12.1494	4.3729	0.3836	0.0086
palmitoyl glycerol 2&1	2-PG	Visual memory	183	-0.0101	0.0242	-0.0303	0.6780	44	0.0640	0.0522	0.1792	0.2287
steareoyl glycerol 2&1	2-SG	Visual memory	181	-0.0030	0.0069	-0.0324	0.6596	44	0.0220	0.0134	0.2328	0.1097
oleoyl glycine	O-Gly	Visual memory	183	-1.9376	1.1778	-0.1275	0.1017	44	4.1018	2.2565	0.2556	0.0774
palmitoyl glycine	P-Gly	Visual memory	183	-0.6747	0.6207	-0.0835	0.2785	44	2.3286	1.2704	0.2468	0.0751
oleoyl glycerol 2&1	2-OG	Abstract reasoning	180	0.1059	0.2310	0.0316	0.6473	46	-0.6703	0.3703	-0.2637	0.0782
linoleoyl serine	L-Ser	Abstract reasoning	184	0.1148	7.6691	0.0010	0.9881	46	41.6109	19.9866	0.3493	0.0441
palmitoyl serine	P-Ser	Abstract reasoning	184	-4.6708	2.0660	-0.1589	0.0250	46	2.7778	3.9699	0.1100	0.4884
linoleic acid	LA	Perceptual organization	172	-0.0002	0.0001	-0.1061	0.1817	44	0.0007	0.0003	0.3103	0.0300

© 2024. Vered S. et al. Published online at http://www.aginganddisease.org/EN/10.14336/AD.2024.1427

linolenic acid	LnA	Perceptual organization	172	-0.0005	0.0002	-0.1521	0.0531	44	0.0017	0.0006	0.3728	0.0099
oleic acid	OA	Perceptual organization	172	-0.0001	0.0001	-0.1135	0.1504	44	0.0003	0.0002	0.2644	0.0596
oleoyl glycerol 2&1	2-OG	Perceptual organization	168	0.0577	0.0303	0.1464	0.0587	44	-0.0997	0.0607	-0.2324	0.1090
linoleoyl glycine	L-Gly	Perceptual organization	171	-0.2485	0.4094	-0.0473	0.5447	44	1.8951	0.8070	0.3275	0.0245
oleoyl glycine	O-Gly	Perceptual organization	172	-0.1969	0.1933	-0.0814	0.3098	44	0.8080	0.3561	0.3029	0.0294
palmitoyl serine	P-Ser	Perceptual organization	172	-0.1760	0.2803	-0.0498	0.5310	44	-1.4715	0.6244	-0.3445	0.0240
arachidonoyl glycerol 2&1	2-AG	Perceptual organization	172	0.0586	0.0745	0.0614	0.4327	44	-0.2203	0.1497	-0.2067	0.1498
arachidonoyl serine	A-Ser	Attention	181	-0.0845	0.0552	-0.1133	0.1274	45	0.2687	0.1057	0.3801	0.0154
linoleic acid	LA	Executive function	176	-0.0003	0.0001	-0.2552	0.0008	44	0.0001	0.0002	0.0878	0.6063
arachidonoyl ethanolamide	AEA	Executive function	175	-0.6372	0.4535	-0.1056	0.1619	44	1.2282	0.8320	0.2420	0.1486
linoleoyl ethanolamide	LEA	Executive function	175	-0.3903	0.2693	-0.1116	0.1492	44	1.1419	0.4835	0.3834	0.0237
oleoyl glycerol 2&1	2-OG	Executive function	172	0.0074	0.0214	0.0260	0.7283	44	-0.0891	0.0363	-0.3727	0.0191
oleoyl glycine	O-Gly	Executive function	176	-0.2954	0.1307	-0.1737	0.0251	44	0.1986	0.2484	0.1322	0.4292
palmitoyl glycine	P-Gly	Executive function	176	-0.1602	0.0694	-0.1756	0.0222	44	0.1186	0.1372	0.1371	0.3932

Cognitive function: Verbal memory = Logical Memory-Delayed Recall test; Visual memory = Visual Reproductions-Delayed Recall test; Abstract reasoning = Similarities test; Perceptual organization = Hooper Visual Organization Test; Attention = Trail-making Test A; Executive function = Trail-making Test B minus A. Models adjusted for age, age squared, sex, education, obesity and time between blood draw and cognitive assessment.

© 2024. Vered S. et al. Published online at http://www.aginganddisease.org/EN/10.14336/AD.2024.1427
